# Prehospital delay and associated factors among stroke patients in Africa: A systematic review and meta-analysis

**DOI:** 10.1371/journal.pone.0326323

**Published:** 2025-06-16

**Authors:** Duguma Debela Ganeti, Amanuel Oljira Dulo, Birhanu Wogane Ilala, Nuritu Bacha Benti, Misganu Diriba, Sadik Abdulwehab, Diriba Etana Tola, Lencho Kajela Solbana

**Affiliations:** 1 Department of Nursing, College of Health Sciences, Assosa University, Assosa, Ethiopia; 2 Department of Midwifery, Institutes of health sciences, Wollega University, Nekemte, Ethiopia; 3 Department of Nursing, Institutes of Health Sciences, Wollega University, Nekemte, Ethiopia; 4 School of Public Health, Wollega University, Nekemte, Ethiopia; Aksaray Education and Research Hospital, TÜRKIYE

## Abstract

**Introduction:**

Stroke is one of the leading causes of death and disability, predominantly affecting low- and middle-income countries. Timely intervention following stroke onset is critical for reducing stroke-related outcomes. However, delayed hospital arrival frequently compromises the effectiveness of treatment. Previous African studies on delayed hospital arrival among stroke patients have reported inconsistent prevalence rates and determinants. Therefore, this systematic review and meta-analysis aimed to assess the overall prevalence of prehospital delay and identify its contributing factors among stroke patients in African countries.

**Methodology:**

This systematic review and meta-analysis followed the Preferred Reporting Items for Systematic Reviews and Meta-Analyses guidelines. The study protocol was registered with PROSPERO under record number CRD42024545741. Relevant studies were retrieved by searching databases such as PubMed, Google Scholar, the Cochrane Library, AJOL, and Hinari. Additional studies were identified through manual searches and the review of article references. The identified studies were critically evaluated for quality. Data were extracted and exported to R (version 4.2.3) and STATA (version 15.0) for analysis. The pooled prevalence of prehospital delay and the pooled odds ratios for associated factors were subsequently estimated. The risk of bias was assessed using a funnel plot and Egger’s test. The results were presented using tables, figures, and statements.

**Results:**

This systematic review and meta-analysis included 16 studies. The pooled prevalence of prehospital delay among stroke patients was 80% (95% CI: 74–86%). Lack of stroke symptom awareness (AOR = 4.43, 95% CI: 1.04–7.83) and increased distance from a health facility (AOR = 1.28, 95% CI: 1.22–1.34) were significantly associated with prehospital delay.

**Conclusion and recommendation:**

The prevalence of prehospital delay among stroke patients in Africa is alarmingly high, with contributing factors including a lack of stroke symptom awareness and increased distance from health facilities. Accordingly, stakeholders should implement targeted interventions that enhance community education on stroke warning signs and alleviate geographic barriers to timely care.

## Introduction

The Global Burden of Disease (GBD) 2021 estimates indicate that stroke is the world’s second leading cause of death, accounting for approximately 7 million deaths each year. Stroke is also the third leading cause of combined death and disability, responsible for over 160 million disability-adjusted life-years (DALYs) lost globally. Nearly 87% of stroke-related deaths and 89% of DALYs occur in low- and middle-income countries [1]. A study in Africa indicated that the three-year mortality rate post-stroke exceeds 80% [2]. The estimated global cost of stroke is over US$890 billion per year, representing 0.66% of the global GDP [1].

Between 1990 and 2021, incident strokes, stroke-related deaths, and DALYs increased by 70%, 44%, and 32%, respectively—and by 2050, these figures are expected to rise further, especially in sub-Saharan Africa. By 2050, global stroke costs could surge to US$880 billion–US$2.31 trillion, primarily affecting low-income countries.[3,4].

Timely treatment following stroke onset can significantly reduce the associated burden [5]. Routine treatments for stroke, including reperfusion therapy for ischemic stroke and immediate reversal of anticoagulation in patients with intracranial hemorrhage, early blood pressure reduction, and other evidence-based interventions for hemorrhagic stroke can lower stroke-related mortality and morbidity when administered promptly [6,7]. However, many patients experience delays in hospital arrival and are unable to receive treatment in a timely manner.

In Africa, evidence shows that only 10% of stroke patients reach the hospital within 3 hours after stroke onset, and over 70% take more than 2 hours to arrive at the hospital [8,9]. Prehospital delay is a primary constraint to cornerstone treatments such as reperfusion therapy and thrombolysis, resulting in less effective treatment options and poorer outcomes [10,11].

Prehospital delay is affected by factors such as transportation type, level of consciousness, initial call destination, motor deficits, NIHSS score, age, symptom awareness, public knowledge, stroke severity, location, and time of onset [12–15]. Research on delayed hospital arrival among stroke patients has primarily focused on developed countries with well-structured socioeconomic, cultural, and health care systems. Studies in Africa on the prevalence of delayed hospital arrival have been inconsistent, and the determinants of delay vary across regions and populations [12,15]. To address this gap, this systematic review and meta-analysis was conducted to assess the overall prevalence and factors associated with delayed hospital admission among stroke patients in Africa.

## Methods and materials

### Protocol registration

This systematic review and meta-analysis was conducted in line with guidelines outlined in the Preferred Reporting Items for Systematic Reviews and Meta-Analysis (PRISMA) [16]. The study protocol was registered in the International Prospective Register of Systematic Reviews (PROSPERO) under registration number CRD42024560119 [17]. A modification regarding the number of authors was made after protocol registration, and this amendment was updated in the registry

### Search strategy and eligibility criteria

A comprehensive search of primary published works on the prevalence and associated factors of prehospital delay among stroke patients available in the English language was conducted using databases such as PubMed, Google, AJOL, Google Scholar, and Hinari. The searches were unrestricted by date and limited to human studies.

Studies available online until June 20, 2024, were identified using free-text terms such as ‘Prevalence,’ ‘Proportion,’ ‘Magnitude,’ ‘Associated factor,’ ‘determinant,’ ‘predictor,’ ‘prehospital delay,’ ‘stroke patients,’ and Medical Subject Heading (MeSH) terms. Additionally, a hand search was performed to identify further studies via Google and by reviewing the reference lists of the identified articles. All identified studies were exported to Mendeley desktop version 1.19.8 and checked for duplicates before undergoing full-text screening and the application of inclusion and exclusion criteria. Duplicates among the identified articles were then removed. Subsequently, four authors (D.D.G, L.K.S., N.B.B, and B.W.I) independently screened the titles and abstracts to determine study relevance, with any discrepancies resolved through discussion with an additional four authors (A.O.D, D.E.T, M.D, and S.W).

### Inclusion and exclusion criteria

The inclusion criteria for full-text articles were: (1) primary observational studies (published or grey literature) assessing the prevalence and/or associated factors of prehospital delay among stroke patients in African countries, and (2) studies available in English. The exclusion criteria were: Clinical trial publications, reviews, abstracts, letters, and conference papers.

### Data extraction

Before conducting data extraction, our review team created a data extraction form using Microsoft Excel. Afterward, three authors collected essential data from the included studies. This data included details such as the first author’s name, publication year, study design, study site, sample size, total cases, associated factors, comparison group, adjusted odds ratio, and 95% CI values for the associated factors.

### Outcomes

The main focus of the study was on the prevalence of prehospital delay and the factors associated with it. Prehospital delay was defined as arriving at the hospital more than 3 or 4.5 hours after stroke onset, depending on the criteria used by the individual studies [13,14,18,19].

### Statistical analysis and presenting findings

Data were first collected using Microsoft Excel and then imported into R (version 4.2.3) and STATA (version 15.0) for analysis. The Meta R package was used to estimate the pooled prevalence of prehospital delay (using Metaprop), and the Metan package in STATA was used to estimate the pooled odds ratio with its 95% CI. In addition, forest and funnel plots were generated using the Meta R package [20].

Cochrane’s Q statistical test of heterogeneity and inverse variance (I²) were used to assess the heterogeneity among the individual studies. The Cochrane’s Q test p‐value < 0.1 and I² ≥ 75% indicate the presence of high heterogeneity among the individual studies [21]. Since significant heterogeneity was observed (P < 0.001), we opted to estimate the pooled prevalence using a weighted average within a random-effects model framework. A forest plot was generated to provide a visual overview of the heterogeneity observed across the studies

Subgroup analyses was carried out to explore variations in delay in hospital arrival according to the country. In addition meta-regression was conducted to identify the source of nonuniformity among the original studies. The forest plots displayed the pooled prevalence of prehospital delay with its 95% CI, the weight of each original study, the number of events (prehospital delay), the total participants, and the proportion of prehospital delay in each original study (Figs 2 and 3). A forest plot was used to display factors that are significantly associated with prehospital delay after pooling the adjusted odds ratios from the included studies (Fig 4).

**Fig 1:**
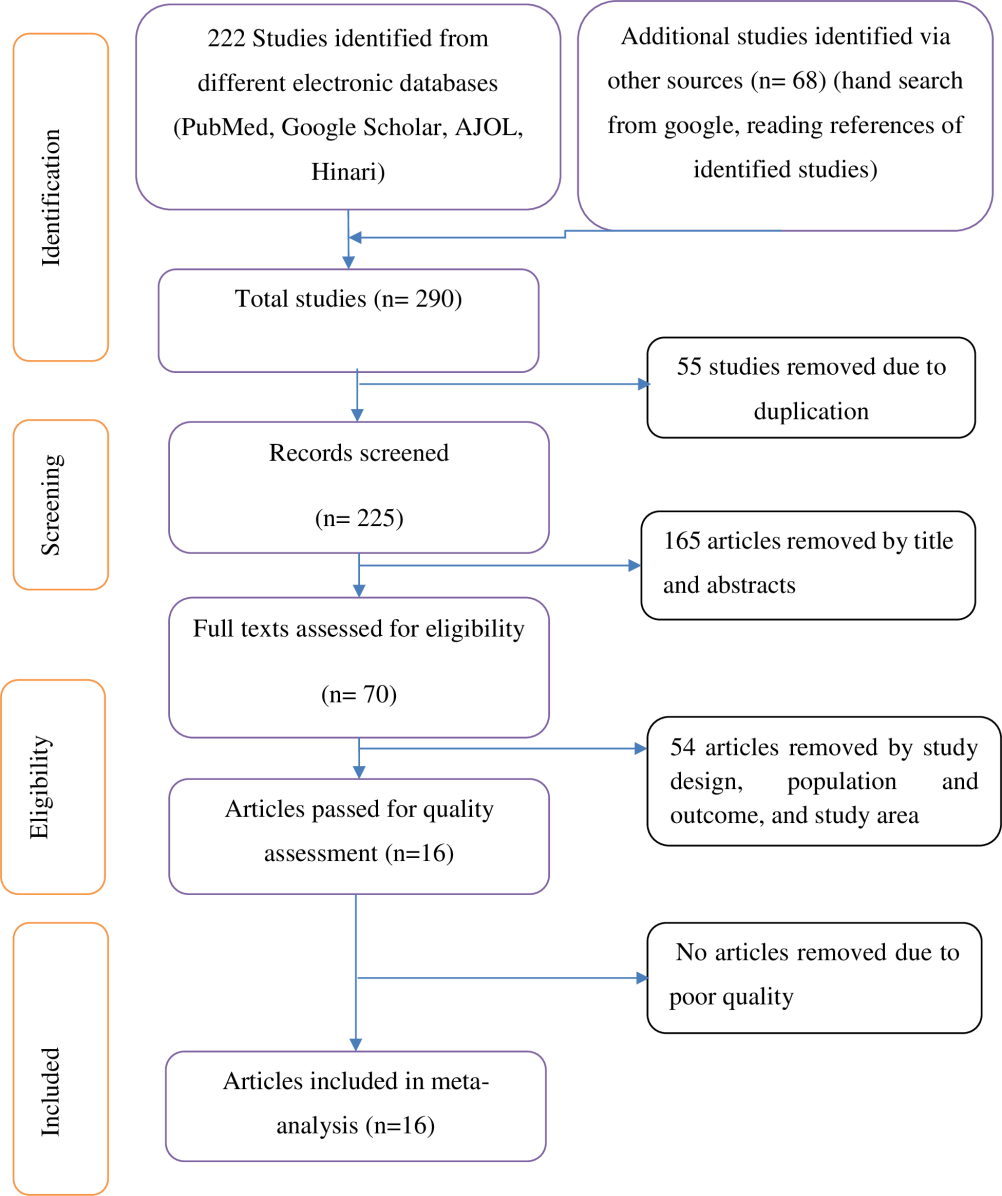
PRISMA flow chart.

**Fig 2:**
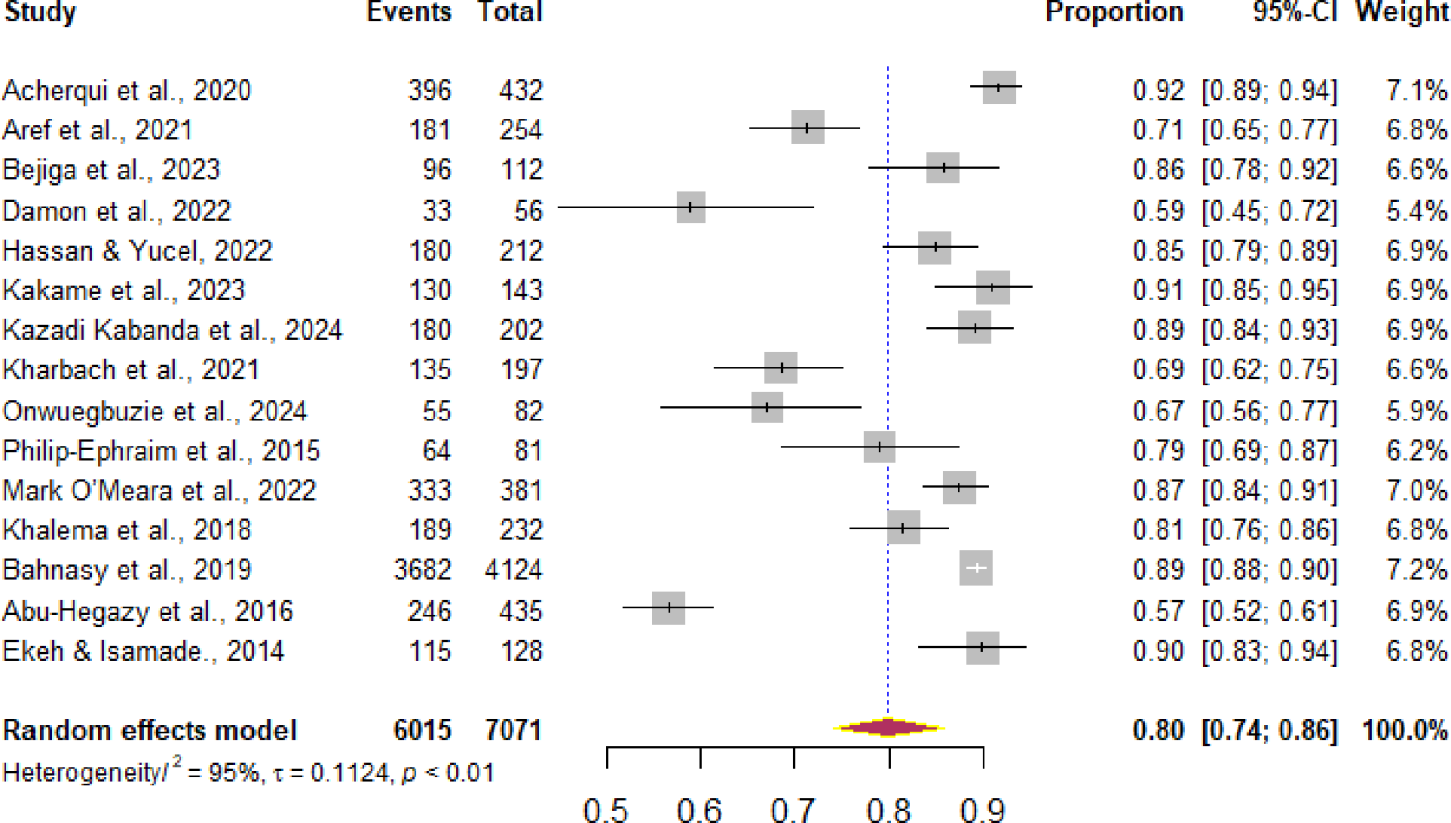
Forest plot indicating the pooled prevalence of prehospital delay in Africa.

**Fig 3:**
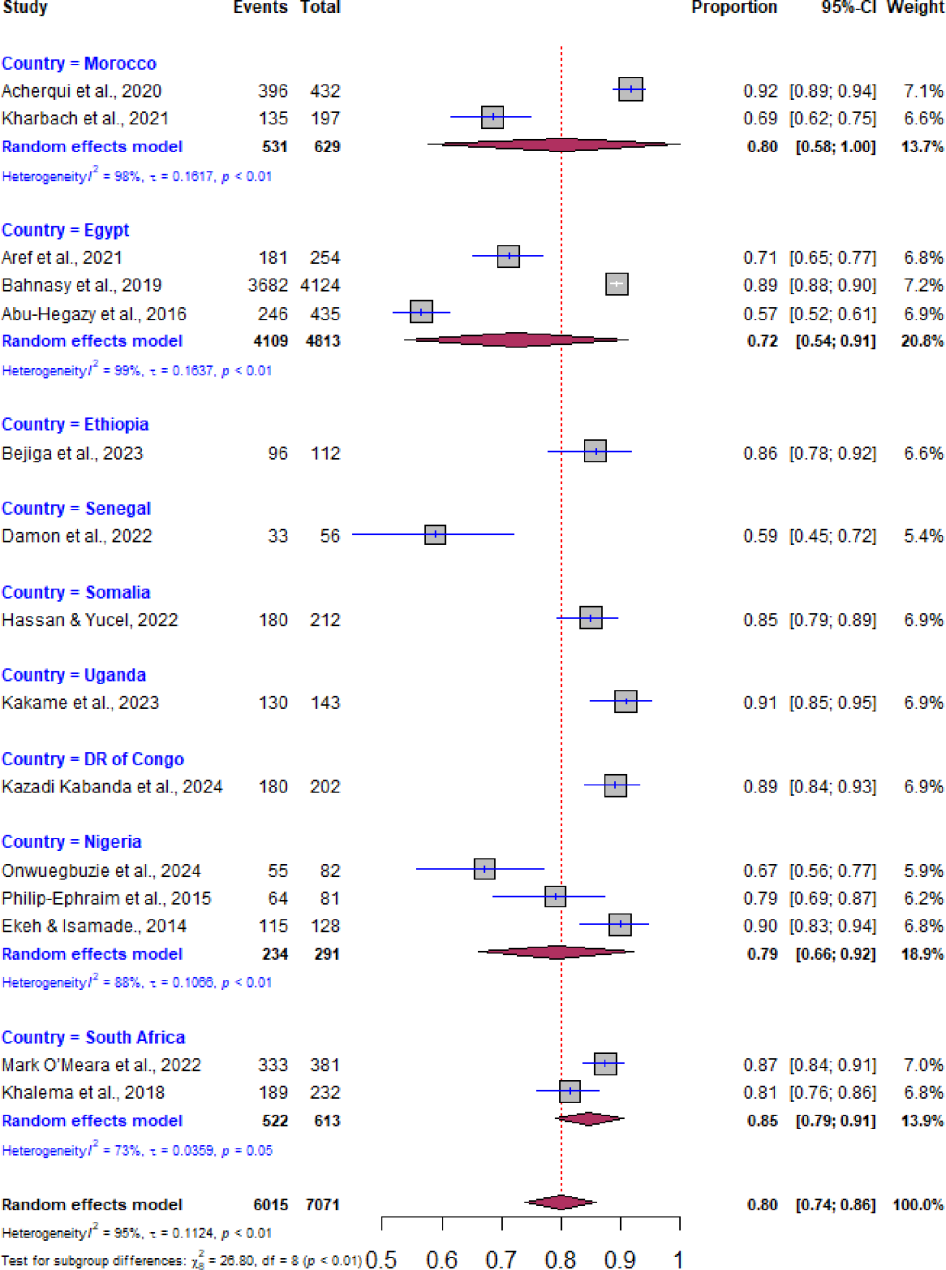
Forest plot indicating sub‐analysis of prehospital delay based on country.

**Fig 4:**
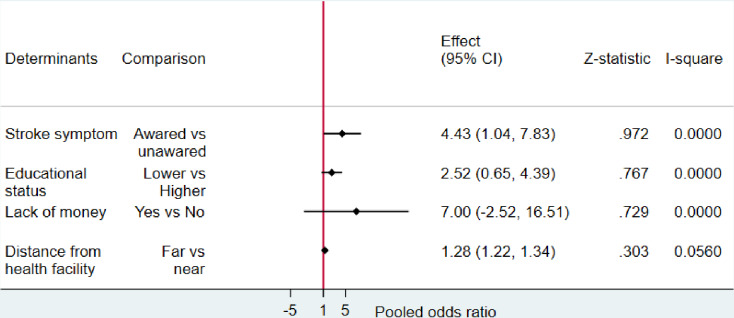
Forest plot indicating the factors associated with prehospital delay.

Additionally, publication bias was examined using a funnel plot and assessed statistically using Egger’s test at the 0.05 significance level. The asymmetry of the funnel plot and the p-value of Egger’s test (< 0.05) indicated the presence of publication bias [22].

Furthermore, a sensitivity analysis was performed to identify the effect of each study on the pooled effect size. Sensitivity analysis was performed by pooling data from 15 studies using the Metaninf function in STATA, which estimated the influence of each study on the overall summary estimate by omitting one study at a time. We presented the characteristics of individual studies and the syntheses using tables, figures, and statements.

## Results

### Study selection

A systematic search of databases (PubMed, Google Scholar, AJOL, and Hinari) initially yielded 222 studies. After excluding 55 duplicate or irrelevant studies, 167 studies remained for title and abstract screening. Upon screening the titles and abstracts, 153 studies did not meet the inclusion criteria, leaving 14 studies eligible for full-text retrieval, all of which met the eligibility criteria.

Additionally, sixty-eight relevant studies were identified by reviewing citations from the identified studies and other sources such as Google. Then after excluding studies due to duplication with studies identified from databases and the unrelated studies, two additional studies were included. Thus, a total of 16 studies, comprising 14 from databases and 2 from other sources, were included in the review (Fig 1).

### Study characteristics

Of the 16 studies, 13 were cross-sectional [19,23–34], one was a prospective cohort study [18], and the remaining two were retrospective chart reviews [35,36]. Two studies were conducted in Morocco [26,32]; three in Nigeria [24,25,28]; three in Egypt [30,31,33]; two in South Africa [35,36]; and one each in Ethiopia [29], Senegal [19], Somalia [27], Democratic Republic of the Congo [18], Uganda [23], and Zimbabwe [34]. Out of the sixteen studies, eight focused exclusively on ischemic stroke [19,26–30,32,33] while another seven included both ischemic and hemorrhagic stroke [18,23–25,28,34,36] and one study covered ischemic and hemorrhagic stroke as well as transient ischemic attack [31].

There was variability across studies in the time thresholds used to classify prehospital delay. Six studies used a threshold of > 4.5 hours [18,26,29,32,35,36], three studies used a threshold of > 4 hours [25,27,31], one study used a threshold of > 3.5 hours [30] and the remaining six used a threshold of > 3 [19,23,24,28,33,34]. Of the 16 studies, 15 were published [18,19,23–28,30–36], while one study was a preprint [29] (Table 1).

**Table 1:** General characteristics of studies included in the meta-analysis.

First author	Study period	Design	Country	Setting	Stroke type	Prehospital delay cutpoints in hour	Event	Total Participant
Acherqui et al., 2020 [32]	January to August 2017	CS	Morocco	Single center	IS	4.5hours	396	432
Aref et al., 2021 [31]	December 7, 2019 and May 10, 2020	CS	Egypt	multi center	IS, HS and TIA	4 hours	181	254
Bejiga et al., 2023 [29]	September 1 to January 30th, 2023	CS	Ethiopia	Single center	AIS	4.5hours	96	112
Damon et al., 2022 [19]	January 1^st^ to June 30th , 2020	CS	Senegal	single center	IS	3 hours	33	56
Hassan & Yucel, 2022 [27]	June 2021 and May 2022	CS	Somalia	Single center	IS	4 hours	180	212
Kakame et al., 2023 [23]	February and April 2022	CS	Uganda	Multi center	IS and HS	3 hours	130	143
Kazadi Kabanda et al., 2024 [18]	May and October 2022	Prospective cohort	Democratic Republic of the Congo	Single center	IS	4.5hours	180	202
Kharbach et al., 2021 [26]	March 2019 to September 2019	CS	Morocco	Single center	IS	4.5hours	135	197
Onwuegbuzie et al., 2024 [25]	March 2022 and July 2023	CS	Nigeria	Single center	IS and HS	4 hours	55	82
Philip-Ephraim et al., 2015 [24]	August 2012– January 2013).	CS	Nigeria	Single center	Both IS and HS	3 hours	64	81
Seremwe et al., 2017 [34]	Not reported	CS	Zimbabwe	Multi center	Both IS and HS	3 hours	Not reported	121
Mark O’Meara et al., 2022 [35]	January 2019 to December 2019	Retrospective review	South Africa	Single center	Ischemic stroke	4.5	333	381
Khalema et al., 2018 [36]	01 January – 31 December 2014.	Retrospective review	South Africa	Single center	Both IS and HS	4.5	189	232
Bahnasy et al., 2019 [30]	November 1, 2017 to October 2018.	CS	Egypt	Multi center	Ischemic stroke	3.5	3682	4124
Abu-Hegazy et al., 2016 [33]	2012-2013	CS	Egypt	Single center	IS	3	246	435
Ekeh & Isamade, 2014 [28]	January – December 2006	CS	Nigeria	Single center	IS and HS	3	115	128

CS = cross sectional.

### The quality score of the study

The quality of the included study was evaluated using the Newcastle‐Ottawa Scale (NOS). Studies having an NOS score of 7 and above were included in the final analysis [37]. (Table 2).

**Table 2:** Newcastle‐Ottawa quality assessment scale of the eligible studies.

S/N	First author	NOS criteria			Total (10)	Remark
		Selection (5)	Comparability (2)	Outcome (3)		
1	Acherqui et al., 2020 [32]	****	–	***	7	Included
2	Aref et al., 2021 [31]	***	**	**	7	Included
3	Bejiga. YD, 2023 [29]	*****	**	**	9	Included
4	Damon MS, 2022 [19]	***	**	***	8	Included
5	Hassan & Yucel, 2022 [27]	*****	**	**	9	Included
6	Kakame et al., 2023 [23]	****	**	**	8	Included
7	KAZADI KABANDA et al., 2024 [18]	****	**	**	8	Included
8	Kharbach et al., 2021 [26]	****	**	**	8	Included
9	Onwuegbuzie et al., 2024 [25]	***	**	**	7	Included
10	Philip-Ephraim et al., 2015 [24]	***	**	**	7	Included
11	Bahnasy et al., 2019 [30]	*****	**	**	9	Included
12	Seremwe et al., 2017 [34]	***	**	**	7	Included
13	Mark O’Meara et al., 2022 [35]	***	**	**	7	Included
14	Khalema et al., 2018 [36]	***	**	**	7	Included
15	Abu-Hegazy et al., 2016 [33]	*****	**	**	9	Included
16	Ekeh & Isamade, 2014 [28]	****	**	**	8	Included

### The pooled prevalence of prehospital delay

In this analysis, fifteen studies involving a total of 6950 participants were included, revealing that 6015 individuals experienced prehospital delay. The pooled prevalence of prehospital delay was found to be 80% (95% CI: 74–86). Significant heterogeneity was observed among the studies, as evidenced by I² of 95% and a p‐value of less than 0.01. Furthermore, a sub-analysis was conducted based on prehospital delays in different countries (Fig 2).

### Sub analysis of prehospital delay

Subgroup analysis was performed based on the countries (Egypt, South Sudan, Nigeria, and Morocco) where the primary studies were conducted. Accordingly, the highest prehospital delay was observed in South Sudan with a prevalence of 86% (95% CI: 79, 91), whereas the lowest delay was observed in Egypt with a prevalence of 72% (95% CI: 54, 91) (Fig 3).

### Factors associated with prehospital delay

Prehospital delay was found to be significantly associated with a lack of stroke symptom awareness (AOR = 4.43, 95% CI: 1.04–7.83) and increased distance from a health facility (AOR = 1.28, 95% CI: 1.22–1.34) (Fig 4).

### Sensitivity analysis

**Fig 5:**
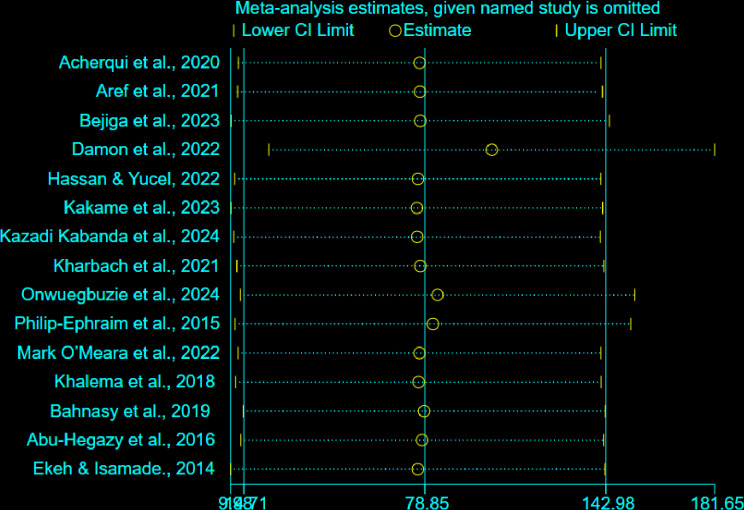
Output of sensitivity analysis.

The sensitivity analysis indicated that omitting any single study did not substantially alter the overall pooled prevalence of delay in hospital arrival. This demonstrates that our pooled estimates are robust and not unduly influenced by any one study (Fig 5).

### Publication bias

**Fig 6:**
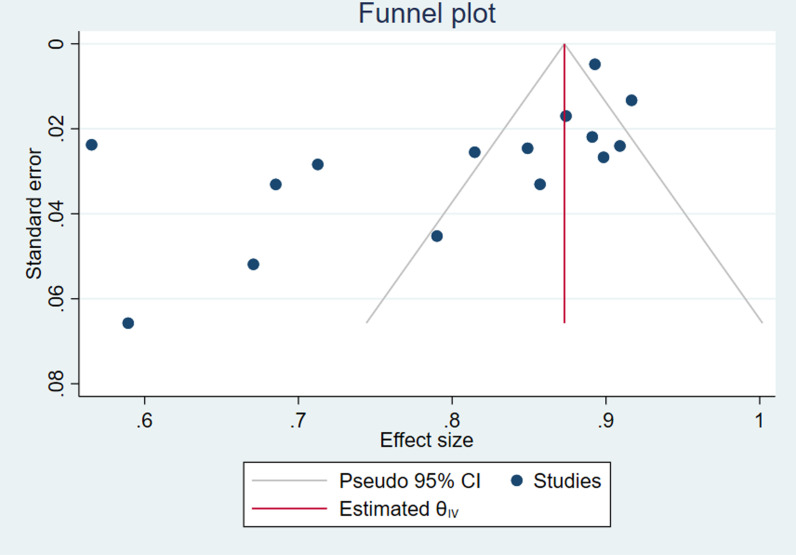
Output of publication bias analysis.

To assess publication bias, we performed Egger’s regression test along with a visual examination of the funnel plot. The results indicated publication bias in the included studies: Egger’s test was statistically significant (p = 0.006) and the funnel plot showed asymmetry (Fig 6). However, after running a trim and fill analysis, no additional studies were imputed. This suggests that although the formal test indicates some bias, its effect is minimal and the original data already provide a balanced view.

## Discussion

This systematic review and meta-analysis study aimed to determine the prevalence and factors associated with prehospital delay among stroke patients in Africa. The pooled prevalence of prehospital delay was found to be 80% (95% CI: 74–86). This prevalence is consistent with primary study findings from countries outside of Africa, such as China, where prehospital delay was observed in 78% and in Brazil, where 71.19% of patients arrived after 3 hours [38,39].

However, this prevalence is higher than findings from developed countries; for example, one Swiss study reported a prehospital delay of only 42% [40], and another study in Switzerland, where prehospital delay was observed in only 22% [41], in the United States of America, the prevalence of prehospital delay was 46.4% [42], and in Thessaloniki, 68.8% of patients arrived within 4.5 hours [43]. These variations in prevalence might be attributed to socioeconomic disparities, differences in the quality of emergency medical services, variations in study methodologies, healthcare accessibility, and the definitions of prehospital delay used. Additionally, low- and middle-income countries like African countries face obstacles like insufficient prehospital services, restricted access to hospital care, and lower use of emergency medical services compared to high-income countries (HICs) [35,44].

This study revealed that the prehospital delay among stroke patients was associated with their level of stroke symptom awareness and the distance from a health facility. Patients who were unaware of stroke signs and symptoms were 4.43 times more likely to experience prehospital delays compared to those who were aware of stroke symptoms. This finding is consistent with similar studies conducted in Switzerland, Spain, China, and Nepal [40,45–47]. This occurs because individuals who are not knowledgeable about the signs and symptoms of a stroke might misinterpret them as indications of less severe issues [34]. Moreover, some patients may mistakenly believe their symptoms will improve on their own. [48]. The results of this systematic review have significant implications for African countries, where many respondents displayed limited knowledge of stroke signs and symptoms [49]. There is a need for educational efforts to enhance awareness of these crucial indicators.

This review also revealed that patients residing in rural or distant areas were 1.28 times more likely to experience prehospital delays compared to those living closer to health facilities. This might be because of the lack of accessible transport by emergency medical services in Africa and those patients found in the distant areas might experience delays when relying on public transportation. However, the study did not identify a significant association between the prehospital delay and the patient’s educational level or lack of money or insurances.

Overall, many stroke patients face delays before receiving medical care, negatively affecting their treatment results and contributing to higher mortality rates and long-term disabilities. This review play a vital role in guiding future research approaches and informing public health policy. It informs the potential areas of improvement for enhancing stroke care in Africa by focusing on secondary prevention strategies to lower stroke-related mortality and morbidity.

This review underscores the need for stroke victims to recognize their symptoms early and for the expansion of stroke care centers to ensure timely treatment, particularly in remote areas. It is imperative for local policymakers to prioritize the incorporation of relevant public health education and acute care for stroke. Additionally, providing education by stroke campaigns to patients, their families and general population about the symptoms of stroke, treatment and the importance of timely arrival at the hospital is very crucial.

Although this study presents valuable insights into the pooled prevalence of prehospital delay and its influencing factors, it is important to acknowledge its limitations. One of the primary constraints is the scarcity of studies included in the analysis of factors affecting hospital presentation time due to variations in the factors studied across different studies. Moreover, the study was confined to 8 African countries, potentially impacting the generalizability of the findings due to substantial variations in prehospital delay influenced by geographical and cultural factors. It is also important to note that this meta-analysis has publication bias, so these findings should be interpreted with caution.

## Supporting information

S1 File List of identified studies. (DOCX)

S2 File Supporting files. (XLSX)

## Abbreviations and acronyms:

PRISMA: Preferred Reporting Items for systematic review and meta-analysis
