# Supplementary material for: Prehospital delay and associated factors among stroke patients in Africa: A systematic review and meta-analysis
Source: PLoS One. 2025 Jun 16;20(6):e0326323. doi: 10.1371/journal.pone.0326323 (PMC12169519; doi:10.1371/journal.pone.0326323)
Supplement: S1 File — (DOCX) [file pone.0326323.s001.docx]

**List of numbered study**

| **S/N** | **First author** | **Remark** |
| --- | --- | --- |
|  |  |  |
|  | (Acherqui *et al.*, 2020) | Included |
|  | (Aref *et al.*, 2021) | Included |
|  | (Bejiga *et al.*, 2023) | Included |
|  | (Damon *et al.*, 2022) | Included |
|  | (Hassan and Yucel, 2022) | Included |
|  | (Kakame *et al.*, 2023) | Included |
|  | (KAZADI KABANDA *et al.*, 2024) | Included |
|  | (Kharbach *et al.*, 2021) | Included |
|  | (Onwuegbuzie, G. A1*, Abdulai, F1, Obianozie, 2024) | Included |
|  | (Philip-Ephraim *et al.*, 2015) | Included |
|  | (Bahnasy, Ragab and Elhassanien, 2019) | Included |
|  | (Seremwe *et al.*, 2017) | Included |
|  | (Mark O’Meara, Ganas and Hendrikse, 2022) | Included |
|  | (Khalema, Diteboho, Lara N. Goldstein, 2018) | Included |
|  | Abu-Hegazy et al., 2016(Abu-hegazy, Elmenshawi and Saad, 2017) | Included |
|  | Ekeh & Isamade, 2014(Ekeh, 2015) | Included |
|  | (Abd-Allah and Moustafa, 2014) | Excluded by title and abstract |
|  | (Addo *et al.*, 2010) | ,, ,, |
|  | (Adeoye *et al.*, 2017) | ,, ,, |
|  | (Adeoye *et al.*, 2019) | ,, ,, |
|  | (Adoukonou *et al.*, 2020) | ,, ,, |
|  | (Akinyemi *et al.*, 2018) | ,, ,, |
|  | (Akinyemi *et al.*, 2021) | ,, ,, |
|  | (Akinyemi *et al.*, 2024) | ,, ,, |
|  | (Akinyemi *et al.*, 2017) | ,, ,, |
|  | (Camlet *et al.*, 2024) | ,, ,, |
|  | (Akpalu *et al.*, 2019) | ,, ,, |
|  | (Albers *et al.*, 2001) | ,, ,, |
|  | (Al-Rukn *et al.*, 2020) | ,, ,, |
|  | (Amarenco *et al.*, 2014) | ,, ,, |
|  | (Assefa *et al.*, 2022) | ,, ,, |
|  | (Benjamin *et al.*, 2017) | ,, ,, |
|  | (Berkowitz *et al.*, 2014) | ,, ,, |
|  | (Bolattürk, 2023) | Excluded by study area |
|  | (Bryer and Wasserman, 2013) | Excluded by title and abstract |
|  | (Camlet *et al.*, 2024) | ,, ,, |
|  | (Chen *et al.*, 2007) | Excluded by study area |
|  | (Citerio and Medicina, 2006) | ,, ,, |
|  | (Cheng *et al.*, 2025) | Excluded by title and abstract |
|  | (Connor, Modi and Warlow, 2007) | ,, ,, |
|  | (Connor, Modi and Warlow, 2009) | ,, ,, |
|  | (Corbett *et al.*, 2022) | ,, ,, |
|  | (Cui, 2016) | ,, ,, |
|  | (Dabilgou *et al.*, 2021) | ,, ,, |
|  | (Damasceno *et al.*, 2010) | ,, ,, |
|  | (Day *et al.*, 2024) | Excluded by study population |
|  | (Deresse and Shaweno, 2015) | Excluded by title and abstract |
|  | (Dieynabou Sow *et al.*, 2016) | ,, ,, |
|  | (Ding *et al.*, 2022) | ,, ,, |
|  | (Dolmans, 2019) | Excluded by methodology |
|  | (Edakkattil *et al.*, 2024) | Excluded by study area |
|  | (Ellis *et al.*, 2018) | Excluded by title and abstract |
|  | (Evenson *et al.*, 2009) | Excluded by methodology |
|  | (Faiz *et al.*, 2012) | Excluded by study area |
|  | (Faiz *et al.*, 2014) | Excluded by title and abstract |
|  | (Fassbender *et al.*, 2013) | Excluded by study area |
|  | (Feigin *et al.*, 2018) | Excluded by title and abstract |
|  | (Fekadu *et al.*, 2020) | ,, ,, |
|  | (Fladt *et al.*, 2019) | Excluded by study area |
|  | (Frohlich, 1990) | Excluded by title and abstract |
|  | (Ghadimi, Hanifi and Dinmohammadi, 2021) | Excluded by study area |
|  | (Gonz, 2019) | Excluded by title and abstract |
|  | (Gonzalez-aquines *et al.*, 2019) | ,, ,, |
|  | (Gude *et al.*, 2023) | ,, ,, |
|  | (Guo *et al.*, 2024) | ,, ,, |
|  | (Gupta *et al.*, 2023) | Excluded by study area |
|  | (Hargreaves, Goodacre and Mortimer, 2014) | Excluded by title and abstract |
|  | (Harrison *et al.*, 2013) | Excluded by title and abstract |
|  | (He *et al.*, 2024) | ,, ,, |
|  | (Heemskerk *et al.*, 2021) | ,, ,, |
|  | (Hoffmann *et al.*, 2000) | ,, ,, |
|  | (Hong *et al.*, 2011) | Excluded by study area |
|  | (Hou *et al.*, 2024) | Excluded by title and abstract |
|  | (Hsia *et al.*, 2011) | Excluded by study area |
|  | (Hur *et al.*, 2011) | ,, ,, |
|  | (Id *et al.*, 2021) | Excluded by title and abstract |
|  | (Imam *et al.*, 2023) | ,, ,, |
|  | (Inatomi, Yonehara and Hashimoto, 2008) | Excluded by title and abstract |
|  | (J R Benger, R Karlsten, 2002) | Excluded by study area |
|  | (Jacqueline Müller-Nordhorn, Karl Wegscheider, Christian H. Nolte, Gerhard J. Jungehülsing, Karin Rossnagel, Andreas Reich; Stephanie Roll, Arno Villringer, 2015) | ,, ,, |
|  | (Jiang *et al.*, 2016) | ,, ,, |
|  | (Jiang *et al.*, 2024) | ,, ,, |
|  | (Jin *et al.*, 2012) | ,, ,, |
|  | (Jones *et al.*, 2012) | Excluded by title and abstract |
|  | (Joubert, 2014) | ,, ,, |
|  | (Kaduka *et al.*, 2019) | ,, ,, |
|  | (Kaduka *et al.*, 2018) | ,, ,, |
|  | (Karen Pitt, 2 Dan-y-Grug, Llanbedr Road, Crickhowell, 2002) | ,, ,, |
|  | (Kendall, 2008) | ,, ,, |
|  | (Kendall, Dutta and Brown, 2015) | ,, ,, |
|  | (Kielkopf *et al.*, 2020) | Excluded by study area |
|  | (Kim *et al.*, 2022) | ,, ,, |
|  | (H. J. Kim *et al.*, 2011) | ,, ,, |
|  | (Y. S. Kim *et al.*, 2011) | ,, ,, |
|  | (Kothari, 1999) | ,, ,, |
|  | (Kobayashi *et al.*, 2007) | Excluded by title and abstract |
|  | (Kpoda *et al.*, 2022) | ,, ,, |
|  | (Krishnamurthi *et al.*, 2014) | ,, ,, |
|  | (Kulein and Sibel, 2014) | Excluded by methodology |
|  | (Kumwenda *et al.*, 2005) | Excluded by title and abstract |
|  | (Kwan *et al.*, 2004) | Excluded by methodology |
|  | (Lachkhem, Rican and Minvielle, 2018) | ,, ,, |
|  | (Lacy *et al.*, 2001) | Excluded by title and abstract |
|  | (Lee, 2018) | ,, ,, |
|  | (Leto *et al.*, 2024) | ,, ,, |
|  | (Lim *et al.*, 2020) | Excluded by study area |
|  | (Limbole, Magne and Lacroix, 2017) | Excluded by title and abstract |
|  | (Lin *et al.*, 2015) | Excluded by study area |
|  | (Longo-Mbenza *et al.*, 2007) | Excluded by title and abstract |
|  | (Lott, Hennes and Dick, 1999) | ,, ,, |
|  | (Maestroni *et al.*, 2008) | Excluded by study area |
|  | (Mainz, Brink and Faurholdt, 2023) | ,, ,, |
|  | (Manawadu, Shuaib and Collas, 2010) | Excluded by title and abstract |
|  | (Mapoure *et al.*, 2019) | ,, ,, |
|  | (Mapoure *et al.*, 2017) | ,, ,, |
|  | (Marta Magrico, Miguel Serôdio, João Nuno Ramos, Rita Ventura, André Sobral-Pinho, João Pedro Marto, 2024) | Excluded by study area |
|  | (Mateen *et al.*, 2021) | Excluded by title and abstract |
|  | (Mathur *et al.*, 2019) | ,, ,, |
|  | (Mcclelland *et al.*, 2015) | ,, ,, |
|  | (Meara, Ganas and Hendrikse, 2022) | ,, ,, |
|  | (Mellon *et al.*, 2016) | ,, ,, |
|  | (Mellon *et al.*, 2013) | ,, ,, |
|  | (Minalloh *et al.*, 2022) | Excluded by study area |
|  | (Morris *et al.*, 2000) | ,, ,, |
|  | (Mould-millman, Sasser and Wallis, 2013) | ,, ,, |
|  | (Nagao *et al.*, 2020) | ,, ,, |
|  | (Napon *et al.*, 2016) | Excluded by title and abstract |
|  | (Nasreldein *et al.*, 2023) | Excluded by population |
|  | (Nepal *et al.*, 2019) | Excluded by study area |
|  | (Nielsen *et al.*, 2012) | Excluded by title and abstract |
|  | (Niklasson, Herlitz and Jood, 2019) | ,, ,, |
|  | (Nkoke *et al.*, 2015) | ,, ,, |
|  | (Nkusi *et al.*, 2017) | ,, ,, |
|  | (Noubiap and Nyaga, 2019) | ,, ,, |
|  | (Ntep-Gweth *et al.*, 2010) | ,, ,, |
|  | (Nutakki *et al.*, 2022) | ,, ,, |
|  | (Ogbole *et al.*, 2015) | ,, ,, |
|  | (Ojagbemi *et al.*, 2021) | ,, ,, |
|  | (Olum *et al.*, 2021) | ,, ,, |
|  | (Ouali *et al.*, 2019) | ,, ,, |
|  | (Owolabi L.F1, 2012) | ,, ,, |
|  | (Owolabi *et al.*, 2019) | ,, ,, |
|  | (Owolabi *et al.*, 2017) | ,, ,, |
|  | (Panagiotis Papapanagiotou a Nicoletta Iacovidou, Konstantinos Spengos, Theodoros Xanthos, Ioannis Zaganas c Afrodite Aggelina, Athanasios Alegakis, 2011) | ,, ,, |
|  | (Patel *et al.*, 2011) | ,, ,, |
|  | (Patel *et al.*, 2005) | ,, ,, |
|  | (Peixoto, Eduardo and Correia, 2017) | Excluded by methodology |
|  | (Perkins *et al.*, 2020) | Excluded by title and abstract |
|  | (Potisopha *et al.*, 2020) | Excluded by methodology |
|  | (Potisopha *et al.*, 2023) | Excluded by study area |
|  | (Per Wester, Johan Rådberg, Bo Lundgren, 1999) | ,, ,, |
|  | (Pulvers and Watson, 2017) | ,, ,, |
|  | (Rajajee and Saver, 2005) | Excluded by title and abstract |
|  | (Rakchue and Poonphol, 2019) | Excluded by study area |
|  | (Ramalan *et al.*, 2023) | Excluded by title and abstract |
|  | (Regenhardt *et al.*, 2019) | ,, ,, |
|  | (Reiff and Michel, 2017) | ,, ,, |
|  | (Rice *et al.*, 2022) | ,, ,, |
|  | (Revathi, Ms and Shankar, 2023) | ,, ,, |
|  | (Roberts and Hughes, 1998) | ,, ,, |
|  | (Robinson *et al.*, 2013) | ,, ,, |
|  | (Rossnagel, 2004) | Excluded by study area |
|  | (Rudd *et al.*, 2015) | Excluded by title and abstract |
|  | (Ruiz *et al.*, 2017) | ,, ,, |
|  | (Rukn *et al.*, 2019) | ,, ,, |
|  | (Sarfo *et al.*, 2015) | ,, ,, |
|  | (Sarfo *et al.*, 2021) | ,, ,, |
|  | (Sarfo, Akassi, Adamu, *et al.*, 2020) | ,, ,, |
|  | (Sarfo, Akassi, Agyei, *et al.*, 2020) | ,, ,, |
|  | (Sarfo, Akassi, *et al.*, 2022) | ,, ,, |
|  | (Sarfo and Ovbiagele, 2020) | ,, ,, |
|  | (Sarfo, Ovbiagele, *et al.*, 2022) | ,, ,, |
|  | (Sarfo *et al.*, 2018) | ,, ,, |
|  | (Shahbandi *et al.*, 2022) | ,, ,, |
|  | (Shaw *et al.*, 2014) | ,, ,, |
|  | (Sheppard *et al.*, 2016) | ,, ,, |
|  | (Sheppard *et al.*, 2013) | ,, ,, |
|  | (Shin *et al.*, 2023) | Excluded by study area |
|  | (Shpak *et al.*, 2019) | Excluded by title and abstract |
|  | (Siddiqui *et al.*, 2008) | Excluded by study area |
|  | (Williams *et al.*, 2009) | ,, ,, |
|  | (Wubshet *et al.*, 2023) | Excluded by title and abstract |
|  | (Xie *et al.*, 2024) | ,, ,, |
|  | (Yanagida *et al.*, 2014b) | Excluded by study area |
|  | (Yang and Hartanto, 2024) | Excluded by lack of outcome of interest |
|  | (Yatsuya *et al.*, 2014) | Excluded by title and abstract |
|  | (YCASiu, TWWong, 1999) | ,, ,, |
|  | (Yoon *et al.*, 2022) | Excluded by study area |
|  | (Yuan *et al.*, 2023) | ,, ,, |
|  | (Yuko Tanaka, Makoto Nakajima, 2009) | ,, ,, |
|  | (Zewdie *et al.*, 2018) | Excluded by title and abstract |
|  | (Zhang *et al.*, 2018) | ,, ,, |
|  | (Zhang *et al.*, 2023) | ,, ,, |
|  | (Siddiqui *et al.*, 2008) | ,, ,, |
|  | (Zhou *et al.*, 2016) | ,, ,, |
|  | (Sheppard *et al.*, 2013) | ,, ,, |
|  | (Smith *et al.*, 2011) | ,, ,, |
|  | (Smyth *et al.*, 2021) | ,, ,, |
|  | (Sommer *et al.*, 2017) | ,, ,, |
|  | (Soto-cámara *et al.*, 2021) | ,, ,, |
|  | (Stead *et al.*, 2008) | ,, ,, |
|  | (Sun *et al.*, 2024) | ,, ,, |
|  | (Rosamond, Gorton and Hinn, no date) | ,, ,, |
|  | (Talle *et al.*, 2023) | ,, ,, |
|  | (Tanne, Yaari and Goldbourt, 1998) | ,, ,, |
|  | (Tataris, Mercer and Govindarajan, 2015) | ,, ,, |
|  | (Teuschl and Ã, 2010) | ,, ,, |
|  | (Tiu, 2022) | ,, ,, |
|  | (Togher, Davy and Siriwardena, 2012) | ,, ,, |
|  | (Tong *et al.*, 2012) | ,, ,, |
|  | (Tran and Mirzaei, 2011) | ,, ,, |
|  | (Tshikwela, Londa and Tongo, 2015) | ,, ,, |
|  | (Ungerer *et al.*, 2020) | ,, ,, |
|  | (Urimubenshi *et al.*, 2018) | ,, ,, |
|  | (V. V. Ashraf, M. Maneesh, R. Praveenkumar, K. Saifudheen, 2015) | ,, ,, |
|  | (Wannarong and Nilanont, 2019) | ,, ,, |
|  | (Williams *et al.*, 2009) | ,, ,, |
|  | (Wubshet *et al.*, 2023) | ,, ,, |
|  | (Xie *et al.*, 2024) | ,, ,, |
|  | (Yanagida *et al.*, 2014a) | ,, ,, |
|  | (Yanagida *et al.*, 2014b) | ,, ,, |
|  | (Yang and Hartanto, 2024) | ,, ,, |
|  | (Yatsuya *et al.*, 2014) | ,, ,, |
|  | (YCASiu, TWWong, 1999) | ,, ,, |
|  | (Yeping Song, Fei Shen, Qing Dong, Liling Wang, 2023) | ,, ,, |

References

Abd-Allah, F. and Moustafa, R.R. (2014) “Burden of stroke in Egypt: Current status and opportunities,” *International Journal of Stroke*, 9(8), pp. 1105–1108. Available at: https://doi.org/10.1111/ijs.12313.

Abu-hegazy, M., Elmenshawi, I. and Saad, M. (2017) “Barriers for acute ischemic stroke treatment using recombinant tissue plasminogen activator in Mansoura Emergency Hospital : prehospital and inhospital delay factors,” pp. 263–267. Available at: https://doi.org/10.4103/1110-1083.202377.

Acherqui, M. *et al.* (2020) “Assessment of eligibility for thrombolysis in acute ischaemic stroke patients in Morocco,” *Pan African Medical Journal*, 36(351), pp. 1–10. Available at: https://doi.org/10.11604/pamj.2020.36.351.22599.

Addo, J. *et al.* (2010) “Delay in Presentation After an Acute Stroke in a Multiethnic Population in South London : The South London Stroke Register,” pp. 1–7. Available at: https://doi.org/10.1161/JAHA.112.001685.

Adeoye, A.M. *et al.* (2017) “Prevalence and Prognostic Features of ECG Abnormalities in Acute Stroke: Findings From the SIREN Study Among Africans.,” *Global heart*, 12(2), pp. 99–105. Available at: https://doi.org/10.1016/j.gheart.2017.01.002.

Adeoye, A.M. *et al.* (2019) “Echocardiographic Abnormalities and Determinants of 1-Month Outcome of Stroke Among West Africans in the SIREN Study.,” *Journal of the American Heart Association*, 8(11), p. e010814. Available at: https://doi.org/10.1161/JAHA.118.010814.

Adoukonou, T. *et al.* (2020) “Prevalence of stroke survivors in Parakou in northern Benin: A door-to-door community survey.,” *Revue neurologique*, 176(10), pp. 839–845. Available at: https://doi.org/10.1016/j.neurol.2020.02.005.

Akinyemi, R. *et al.* (2017) “Interleukin-6 (IL-6) rs1800796 and cyclin dependent kinase inhibitor (CDKN2A/CDKN2B) rs2383207 are associated with ischemic stroke in indigenous West African Men.,” *Journal of the neurological sciences*, 379, pp. 229–235. Available at: https://doi.org/10.1016/j.jns.2017.05.046.

Akinyemi, R. *et al.* (2018) “APOL1, CDKN2A/CDKN2B, and HDAC9 polymorphisms and small vessel ischemic stroke.,” *Acta neurologica Scandinavica*, 137(1), pp. 133–141. Available at: https://doi.org/10.1111/ane.12847.

Akinyemi, R.O. *et al.* (2021) “Stroke in Africa: profile, progress, prospects and priorities,” *Nature Reviews Neurology*, 17(10), pp. 634–656. Available at: https://doi.org/10.1038/s41582-021-00542-4.

Akinyemi, R.O. *et al.* (2024) “Novel functional insights into ischemic stroke biology provided by the first genome-wide association study of stroke in indigenous Africans.,” *Genome medicine*, 16(1), p. 25. Available at: https://doi.org/10.1186/s13073-023-01273-5.

Akpalu, A. *et al.* (2019) “Differential Impact of Risk Factors on Stroke Occurrence Among Men Versus Women in West Africa.,” *Stroke*, 50(4), pp. 820–827. Available at: https://doi.org/10.1161/STROKEAHA.118.022786.

Al-Rukn, S. *et al.* (2020) “Stroke in the Middle-East and North Africa: A 2-year prospective observational study of intravenous thrombolysis treatment in the region. Results from the SITS-MENA Registry.,” *International journal of stroke : official journal of the International Stroke Society*, 15(9), pp. 980–987. Available at: https://doi.org/10.1177/1747493019874729.

Albers, G.W. *et al.* (2001) “Aptiganel hydrochloride in acute ischemic stroke: a randomized controlled trial.,” *JAMA*, 286(21), pp. 2673–2682. Available at: https://doi.org/10.1001/jama.286.21.2673.

Amarenco, P. *et al.* (2014) “Impact of living and socioeconomic characteristics on cardiovascular risk in ischemic stroke patients.,” *International journal of stroke : official journal of the International Stroke Society*, 9(8), pp. 1065–1072. Available at: https://doi.org/10.1111/ijs.12290.

Aref, H.M. *et al.* (2021) “Pre-hospital causes for delayed arrival in acute ischemic stroke before and during the COVID-19 pandemic: A study at two stroke centers in Egypt,” *PLoS ONE*, 16(7 July), pp. 1–11. Available at: https://doi.org/10.1371/journal.pone.0254228.

Assefa, M. *et al.* (2022) “Factors associated with stroke associated pneumonia among adult stroke patients admitted to university of Gondar hospital, Northwest Ethiopia.,” *Scientific reports*, 12(1), p. 12724. Available at: https://doi.org/10.1038/s41598-022-14656-2.

Bahnasy, W.S., Ragab, O.A.A. and Elhassanien, M.E. (2019) “Stroke onset to needle delay: Where these golden hours are lost? An Egyptian center experience,” *eNeurologicalSci*, 14(December 2018), pp. 68–71. Available at: https://doi.org/10.1016/j.ensci.2019.01.003.

Bejiga, Y.D. *et al.* (2023) “Assessment of hospital arrival time and associated factors of patients with acute ischemic stroke to introduce thrombolytic therapy at Jimma Medical,” pp. 1–17.

Benjamin, L.A. *et al.* (2017) “The Role of Human Immunodeficiency Virus-Associated Vasculopathy in the Etiology of Stroke.,” *The Journal of infectious diseases*, 216(5), pp. 545–553. Available at: https://doi.org/10.1093/infdis/jix340.

Berkowitz, A.L. *et al.* (2014) “Aspirin for acute stroke of unknown etiology in resource-limited settings: a decision analysis.,” *Neurology*, 83(9), pp. 787–793. Available at: https://doi.org/10.1212/WNL.0000000000000730.

Bolattürk, Ö.F. (2023) “Factors affecting the presentation time of patients with acute stroke to hospital and level of awareness of thrombolytic therapy,” 6(1), pp. 77–81. Available at: https://doi.org/10.32322/jhsm.1177076.

Bryer, A. and Wasserman, S. (2013) “Thrombolysis for acute ischemic stroke in South Africa.,” *International journal of stroke : official journal of the International Stroke Society*, 8 Suppl A1, pp. 112–113. Available at: https://doi.org/10.1111/ijs.12059.

Camlet, K. *et al.* (2024) “Burden of ischemic stroke in sub-Saharan African countries based on DALYs index - trend analysis and identification of risk factors.,” *Przeglad epidemiologiczny*, 78(2), pp. 193–206. Available at: https://doi.org/10.32394/pe/190845.

Chen, C. *et al.* (2007) “P RE - HOSPITAL AND I N - HOSPITAL D ELAYS A FTER O NSET OF A CUTE I SCHEMIC S TROKE — A H OSPITAL - BASED S TUDY IN S OUTHERN T AIWAN,” 23(11).

Cheng, F. *et al.* (2025) “Epidemiological trends and age-period-cohort effects on ischemic stroke burden across the BRICS-plus from 1992 to 2021.,” *BMC public health*, 25(1), p. 137. Available at: https://doi.org/10.1186/s12889-025-21310-9.

Citerio, G. and Medicina, D. (2006) “Early stroke care in Italy—a steep way ahead: an observational study,” pp. 608–612. Available at: https://doi.org/10.1136/emj.2005.032219.

Connor, M.D., Modi, G. and Warlow, C.P. (2007) “Accuracy of the Siriraj and Guy’s Hospital Stroke Scores in urban South Africans.,” *Stroke*, 38(1), pp. 62–68. Available at: https://doi.org/10.1161/01.STR.0000251853.62387.68.

Connor, M.D., Modi, G. and Warlow, C.P. (2009) “Differences in the nature of stroke in a multiethnic urban South African population: the Johannesburg hospital stroke register.,” *Stroke*, 40(2), pp. 355–362. Available at: https://doi.org/10.1161/STROKEAHA.108.521609.

Corbett, C. *et al.* (2022) “Prevalence and Characteristics of HIV-Associated Stroke in a Tertiary Hospital Setting in South Africa.,” *Neurology*, 99(9), pp. e904–e915. Available at: https://doi.org/10.1212/WNL.0000000000200780.

Cui, T. (2016) “MTHFR C677T mutation increased the risk of Ischemic Stroke, especially in large-artery atherosclerosis in adults: an updated meta-analysis from 38 researches.,” *The International journal of neuroscience*, 126(1), pp. 10–19. Available at: https://doi.org/10.3109/00207454.2014.990559.

Dabilgou, A.A. *et al.* (2021) “Frequency of recurrent stroke in Burkina Faso: an observational hospital based study of 6 months.,” *The Pan African medical journal*, 40, p. 108. Available at: https://doi.org/10.11604/pamj.2021.40.108.23098.

Damasceno, A. *et al.* (2010) “An epidemiological study of stroke hospitalizations in Maputo, Mozambique: a high burden of disease in a resource-poor country.,” *Stroke*, 41(11), pp. 2463–2469. Available at: https://doi.org/10.1161/STROKEAHA.110.594275.

Damon, M.A.S. *et al.* (2022) “Pre-hospital delay in patients with ischemic stroke in the Fann Teaching Hospital, Dakar, Senegal in 2020,” *Pan African Medical Journal*, 41. Available at: https://doi.org/10.11604/pamj.2022.41.79.30191.

Day, J. *et al.* (2024) “Healthcare professional views about a prehospital redirection pathway for stroke thrombectomy : a multiphase deductive qualitative study,” pp. 429–435. Available at: https://doi.org/10.1136/emermed-2023-213350.

Deresse, B. and Shaweno, D. (2015) “Epidemiology and in-hospital outcome of stroke in South Ethiopia.,” *Journal of the neurological sciences*, 355(1–2), pp. 138–142. Available at: https://doi.org/10.1016/j.jns.2015.06.001.

Dieynabou Sow, A. *et al.* (2016) “Prognosis of spontaneous hemorrhagic stroke in people under 55 in Senegal, a developing country in Africa: a series of 53 cases.,” *Medecine et sante tropicales*, 26(2), pp. 170–174. Available at: https://doi.org/10.1684/mst.2016.0542.

Ding, Q. *et al.* (2022) “Global, Regional, and National Burden of Ischemic Stroke, 1990-2019.,” *Neurology*, 98(3), pp. e279–e290. Available at: https://doi.org/10.1212/WNL.0000000000013115.

Dolmans, L.S. (2019) “Patient delay in TIA : a systematic review,” *Journal of Neurology*, 266(5), pp. 1051–1058. Available at: https://doi.org/10.1007/s00415-018-8977-6.

Edakkattil, S. *et al.* (2024) “Prehospital Factors Associated with Delayed Hospital Arrival of Stroke Patients : A Regional Single ‑ Center Study from India,” pp. 165–172. Available at: https://doi.org/10.4103/aian.aian.

Ekeh, B.C. (2015) “Time of presentation of stroke patients in a tertiary hospital in northern Nigeria , west africa,” (January 2014), pp. 1–4.

Ellis, J.P. *et al.* (2018) “Ischemic stroke as a complication of cryptococcal meningitis and immune reconstitution inflammatory syndrome: a case report.,” *BMC infectious diseases*, 18(1), p. 520. Available at: https://doi.org/10.1186/s12879-018-3386-0.

Evenson, K.R.Ã. *et al.* (2009) “Topical review A comprehensive review of prehospital and in-hospital delay times in acute stroke care,” 4(June), pp. 187–199.

Faiz, K.W. *et al.* (2012) “Prehospital delay in acute stroke and TIA,” (August 2016). Available at: https://doi.org/10.1136/emermed-2012-201543.

Faiz, K.W. *et al.* (2014) “Factors Related to Decision Delay in Acute Stroke,” *Journal of Stroke and Cerebrovascular Diseases*, 23(3), pp. 534–539. Available at: https://doi.org/10.1016/j.jstrokecerebrovasdis.2013.05.007.

Fassbender, K. *et al.* (2013) “Streamlining of prehospital stroke management : the golden hour,” *The Lancet Neurology*, 12(6), pp. 585–596. Available at: https://doi.org/10.1016/S1474-4422(13)70100-5.

Feigin, V.L. *et al.* (2018) “Global, Regional, and Country-Specific Lifetime Risks of Stroke, 1990 and 2016.,” *The New England journal of medicine*, 379(25), pp. 2429–2437. Available at: https://doi.org/10.1056/NEJMoa1804492.

Fekadu, G. *et al.* (2020) “Clinical characteristics and treatment outcomes among stroke patients hospitalized to Nekemte referral hospital, western Ethiopia.,” *Journal of clinical neuroscience : official journal of the Neurosurgical Society of Australasia*, 71, pp. 170–176. Available at: https://doi.org/10.1016/j.jocn.2019.08.075.

Fladt, J. *et al.* (2019) “Reasons for Prehospital Delay in Acute Ischemic Stroke,” *Journal of the American Heart Association*, 8(20). Available at: https://doi.org/10.1161/JAHA.119.013101.

Frohlich, E.P. (1990) “Vascular complications in women using the low steroid content combined oral contraceptive pills: case reports and review of the literature.,” *Obstetrical & gynecological survey*, 45(9), pp. 578–584. Available at: https://doi.org/10.1097/00006254-199009000-00003.

Ghadimi, N., Hanifi, N. and Dinmohammadi, M. (2021) “Factors Affecting Pre-Hospital and In-Hospital Delays in Treatment of Ischemic Stroke ; a Prospective Cohort Study,” 9(1), pp. 1–9.

Gonz, J. (2019) “Factors Associated with Shortening of Prehospital Delay among Patients with Acute Ischemic Stroke.”

Gonzalez-aquines, A. *et al.* (2019) “ARTICLE IN PRESS Contribution of Onset-to-Alarm Time to Prehospital Delay in Patients with Ischemic Stroke,” pp. 1–6. Available at: https://doi.org/10.1016/j.jstrokecerebrovasdis.2019.104331.

Gude, M.F. *et al.* (2023) “Associations between emergency call stroke triage and pre ‑ hospital delay , primary hospital admission , and acute reperfusion treatment among early comers with acute ischemic stroke,” *Internal and Emergency Medicine*, 18(8), pp. 2355–2365. Available at: https://doi.org/10.1007/s11739-023-03349-x.

Guo, X. *et al.* (2024) “Global burden of ischemic stroke attributable to high body mass index in 204 countries and territories, 1990-2021.,” *BMC cardiovascular disorders*, 24(1), p. 584. Available at: https://doi.org/10.1186/s12872-024-04259-2.

Gupta, A.K. *et al.* (2023) “Causes of Pre-hospital Delay in Acute Stroke in Punjab,” 15(5). Available at: https://doi.org/10.7759/cureus.39180.

Hargreaves, K., Goodacre, S. and Mortimer, P. (2014) “Paramedic perceptions of the feasibility and practicalities of prehospital clinical trials : a questionnaire survey,” pp. 499–504. Available at: https://doi.org/10.1136/emermed-2013-202346.

Harrison, M. *et al.* (2013) “Patients ’ and carers ’ experiences of gaining access to acute stroke care : a qualitative study,” pp. 1033–1037. Available at: https://doi.org/10.1136/emermed-2012-201974.

Hassan, M.S. and Yucel, Y. (2022) “Factors Influencing Early Hospital Arrival of Patients with Acute Ischemic Stroke, Cross-Sectional Study at Teaching Hospital in Mogadishu Somalia,” *Journal of Multidisciplinary Healthcare*, 15, pp. 2891–2899. Available at: https://doi.org/10.2147/JMDH.S392922.

He, Q. *et al.* (2024) “Global, Regional, and National Burden of Stroke, 1990-2021: A Systematic Analysis for Global Burden of Disease 2021.,” *Stroke*, 55(12), pp. 2815–2824. Available at: https://doi.org/10.1161/STROKEAHA.124.048033.

Heemskerk, J.L. *et al.* (2021) “Time Is Brain : Prehospital Emergency Medical,” *Mayo Clinic Proceedings*, 96(6), pp. 1446–1457. Available at: https://doi.org/10.1016/j.mayocp.2020.08.050.

Hoffmann, M. *et al.* (2000) “Cerebrovascular disease in young, HIV-infected, black Africans in the KwaZulu Natal province of South Africa.,” *Journal of neurovirology*, 6(3), pp. 229–236. Available at: https://doi.org/10.3109/13550280009015825.

Hong, E.S. *et al.* (2011) “Factors associated with prehospital delay in acute stroke,” pp. 790–794. Available at: https://doi.org/10.1136/emj.2010.094425.

Hou, S. *et al.* (2024) “Global, regional, and national epidemiology of ischemic stroke from 1990 to 2021.,” *European journal of neurology*, 31(12), p. e16481. Available at: https://doi.org/10.1111/ene.16481.

Hsia, A.W. *et al.* (2011) “Understanding Reasons for Delay in Seeking Acute Stroke Care in an Underserved Urban Population.” Available at: https://doi.org/10.1161/STROKEAHA.110.604736.

Hur, J.W. *et al.* (2011) “Factors Influencing Prehospital and Inhospital Time Delays for Ischemic Stroke Patients,” 22(3), pp. 193–199.

Id, E.L. *et al.* (2021) “Impact of onset-to-door time on outcomes and factors associated with late hospital arrival in patients with acute ischemic stroke,” pp. 1–12. Available at: https://doi.org/10.1371/journal.pone.0247829.

Imam, Y.Z. *et al.* (2023) “Rescue Stent Placement for Acute Ischemic Stroke with Large Vessel Occlusion Refractory to Mechanical Thrombectomy: A Multiethnic Middle Eastern/African/Asian Cohort.,” *Journal of vascular and interventional radiology : JVIR*, 34(10), pp. 1740–1748. Available at: https://doi.org/10.1016/j.jvir.2023.06.005.

Inatomi, Y., Yonehara, T. and Hashimoto, Y. (2008) “Pre-hospital delay in the use of intravenous rt-PA for acute ischemic stroke in Japan,” 270, pp. 127–132. Available at: https://doi.org/10.1016/j.jns.2008.02.018.

J R Benger, R Karlsten, B.E. (2002) “Prehospital thrombolysis : lessons from Sweden and their application to the United Kingdom,” pp. 578–584.

Jacqueline Müller-Nordhorn, Karl Wegscheider, Christian H. Nolte, Gerhard J. Jungehülsing, Karin Rossnagel, Andreas Reich; Stephanie Roll, Arno Villringer, and S.N.W. (2015) “Population-Based Intervention to Reduce Prehospital Delays in Patients With Cerebrovascular Events,” 169(16), pp. 1484–1490.

Jiang, B. *et al.* (2016) “Pre-hospital delay and its associated factors in first-ever stroke registered in communities from three cities in China,” *Scientific Reports*, 6(February), pp. 1–11. Available at: https://doi.org/10.1038/srep29795.

Jiang, Y. *et al.* (2024) “Patient Education and Counseling Analyzing prehospital delays in recurrent acute ischemic stroke : Insights from interpretable machine learning,” *Patient Education and Counseling*, 123(February), p. 108228. Available at: https://doi.org/10.1016/j.pec.2024.108228.

Jin, H. *et al.* (2012) “Factors Associated With Prehospital Delays in the Presentation of Acute Stroke in Urban China,” (8), pp. 362–370. Available at: https://doi.org/10.1161/STROKEAHA.111.623512.

Jones, S.P. *et al.* (2012) “Callers ’ experiences of making emergency calls at the onset of acute stroke : a qualitative study,” pp. 2010–2013. Available at: https://doi.org/10.1136/emj.2010.108563.

Joubert, G. (2014) “Computed tomography stroke findings and population demographics at Pelonomi Hospital , Bloemfontein,” pp. 1–6.

Kaduka, L. *et al.* (2018) “Stroke Mortality in Kenya’s Public Tertiary Hospitals: A Prospective Facility-Based Study.,” *Cerebrovascular diseases extra*, 8(2), pp. 70–79. Available at: https://doi.org/10.1159/000488205.

Kaduka, L. *et al.* (2019) “Disability-Adjusted Life-Years Due to Stroke in Kenya.,” *Neuroepidemiology*, 53(1–2), pp. 48–54. Available at: https://doi.org/10.1159/000498970.

Kakame, K.T. *et al.* (2023) “Prevalence and factors associated with pre-hospital delay among acute stroke patients at Mulago and Kiruddu national referral hospitals, Kampala: a cross-sectional study,” *BMC Neurology*, 23(1), pp. 1–12. Available at: https://doi.org/10.1186/s12883-023-03413-1.

Karen Pitt, 2 Dan-y-Grug, Llanbedr Road, Crickhowell, P.N. 1DD (2002) “Prehospital selection of patients for thrombolysis by paramedics,” pp. 260–264.

KAZADI KABANDA, I. *et al.* (2024) “Stroke signs knowledge and factors associated with a delayed hospital arrival of patients with acute stroke in Kinshasa,” *Heliyon*, 10(7). Available at: https://doi.org/10.1016/j.heliyon.2024.e28311.

Kendall, J. (2008) “Thrombolysis for acute ischaemic stroke : a new challenge for emergency medicine,” pp. 471–476. Available at: https://doi.org/10.1136/emj.2007.054668.

Kendall, J., Dutta, D. and Brown, E. (2015) “Reducing delay to stroke thrombolysis — lessons learnt from the Stroke 90 Project,” pp. 100–104. Available at: https://doi.org/10.1136/emermed-2013-202993.

Khalema, Diteboho, Lara N. Goldstein, S.L. (2018) “A retrospective analysis of time delays in patients presenting with stroke to an academic emergency department,” *South African Journal of Radiology*, 22(1), pp. 1–6. Available at: https://doi.org/10.4102/sajr.v22i1.1319.

Kharbach, A. *et al.* (2021) “Ischemic stroke in Morocco: Prehospital delay and associated factors,” *Revue d’Epidemiologie et de Sante Publique*, 69(6), pp. 345–359. Available at: https://doi.org/10.1016/j.respe.2021.03.010.

Kielkopf, M. *et al.* (2020) “Temporal trends and risk factors for delayed hospital admission in suspected stroke patients,” *Journal of Clinical Medicine*, 9(8), pp. 1–8. Available at: https://doi.org/10.3390/jcm9082376.

Kim, D. *et al.* (2022) “Pre-Hospital Delay in Patients With Acute Stroke During the Initial Phase of the Coronavirus Disease 2019 Outbreak,” 37(6), pp. 1–12.

Kim, H.J. *et al.* (2011) “Selected Topics : Prehospital Care FACTORS ASSOCIATED WITH PREHOSPITAL DELAY FOR ACUTE STROKE IN,” *JEM*, 41(1), pp. 59–63. Available at: https://doi.org/10.1016/j.jemermed.2010.04.001.

Kim, Y.S. *et al.* (2011) “Stroke awareness decreases prehospital delay after acute ischemic stroke in korea.”

Kobayashi, A. *et al.* (2007) “Lack of experience of intravenous thrombolysis for acute ischaemic stroke does not influence the proportion of patients treated,” pp. 96–100. Available at: https://doi.org/10.1136/emj.2006.040204.

Kothari, R. (1999) “Acute Stroke " Delays to Presentation and Emergency Department Evaluation,” (January), pp. 3–8.

Kpoda, H.B.N. *et al.* (2022) “Prognostic Factors of the Lethality of Stroke at the Sourô Sanou University Teaching Hospital of Burkina Faso.,” *Cerebrovascular diseases extra*, 12(1), pp. 36–46. Available at: https://doi.org/10.1159/000523888.

Krishnamurthi, R. V *et al.* (2014) “The global burden of hemorrhagic stroke: a summary of findings from the GBD 2010 study.,” *Global heart*, 9(1), pp. 101–106. Available at: https://doi.org/10.1016/j.gheart.2014.01.003.

Kulein, E. and Sibel, K. (2014) “Factors associated with early hospital arrival in acute ischemic stroke patients,” pp. 10–14. Available at: https://doi.org/10.1007/s10072-014-1796-3.

Kumwenda, J.J. *et al.* (2005) “Differential diagnosis of stroke in a setting of high HIV prevalence in Blantyre, Malawi.,” *Stroke*, 36(5), pp. 960–964. Available at: https://doi.org/10.1161/01.STR.0000162585.97216.ef.

Kwan, J. *et al.* (2004) “A systematic review of barriers to delivery of thrombolysis for acute stroke,” 33(2), pp. 116–121. Available at: https://doi.org/10.1093/ageing/afh064.

Lachkhem, Y., Rican, S. and Minvielle, É. (2018) “Understanding delays in acute stroke care : a systematic review of reviews,” *European Journal of Public Health*, 28(3), pp. 426–433. Available at: https://doi.org/10.1093/eurpub/cky066.

Lacy, C.R. *et al.* (2001) “Stroke Time Registry for Outcomes Knowledge and,” pp. 63–69. Available at: https://doi.org/10.1161/01.STR.32.1.63.

Lee, S.J. (2018) “Association between Prehospital Delay Status and Stroke Severity in Acute Ischemic Stroke : Shift-Analysis Approach,” 7(2). Available at: https://doi.org/10.4172/2167-1168.1000452.

Leto, N. *et al.* (2024) “Prehospital identification of acute ischaemic stroke with large vessel occlusion : a retrospective study from western Norway,” pp. 249–254. Available at: https://doi.org/10.1136/emermed-2023-213236.

Lim, S.H. *et al.* (2020) “Factors related to prehospital delay and decision delay among acute stroke patients in a district hospital , Malaysia,” pp. 241–249.

Limbole, E.B., Magne, J. and Lacroix, P. (2017) “Stroke characterization in Sun Saharan Africa: Congolese population.,” *International journal of cardiology*, 240, pp. 392–397. Available at: https://doi.org/10.1016/j.ijcard.2017.04.063.

Lin, C. *et al.* (2015) “Prehospital delay and emergency department management of ischemic stroke patients in taiwan, r.o.c.,” 3127(September). Available at: https://doi.org/10.1080/10903129908958936.

Longo-Mbenza, B. *et al.* (2007) “Relationship between waist circumference and cholesterol in Central Africans with congestive heart failure.,” *West African journal of medicine*, 26(3), pp. 183–190. Available at: https://doi.org/10.4314/wajm.v26i3.28306.

Lott, C., Hennes, H.J. and Dick, W. (1999) “Stroke a medical emergency L-,” pp. 2–8.

Maestroni, A. *et al.* (2008) “Factors influencing delay in presentation for acute stroke in an emergency department in Milan , Italy,” pp. 340–345. Available at: https://doi.org/10.1136/emj.2007.048389.

Mainz, J., Brink, J. and Faurholdt, M. (2023) “Treatment Delays and Chance of Reperfusion Therapy in Patients with Acute Stroke : A Danish Nationwide Study,” pp. 275–282. Available at: https://doi.org/10.1159/000526733.

Manawadu, D., Shuaib, A. and Collas, D.M. (2010) “Emergency department or general practitioner following transient ischaemic attack ? A comparison of patient behaviour and speed of assessment in England and Canada,” pp. 364–368. Available at: https://doi.org/10.1136/emj.2009.074831.

Mapoure, Y.N. *et al.* (2017) “Gender-Related Differences and Short-Term Outcome of Stroke: Results from a Hospital-Based Registry in Sub-Saharan Africa.,” *Neuroepidemiology*, 49(3–4), pp. 179–188. Available at: https://doi.org/10.1159/000484319.

Mapoure, Y.N. *et al.* (2019) “Cardio-embolic stroke: Lessons from a single centre in Sub-Saharan Africa.,” *Revue neurologique*, 175(9), pp. 544–551. Available at: https://doi.org/10.1016/j.neurol.2019.02.004.

Mark O’Meara, R., Ganas, U. and Hendrikse, C. (2022) “Access to acute stroke care: A retrospective descriptive analysis of stroke patients’ journey to a district hospital,” *African Journal of Emergency Medicine*, 12(4), pp. 366–372. Available at: https://doi.org/10.1016/j.afjem.2022.07.010.

Marta Magrico, Miguel Serôdio, João Nuno Ramos, Rita Ventura, André Sobral-Pinho, João Pedro Marto, and M.V.-B. (2024) “Are we missing an opportunity? Prehospital delay in patients with acute ischemic stroke and known atrial fibrillatio,” 43, pp. 321–325.

Mateen, F.J. *et al.* (2021) “Measuring Ambulation, Motor, and Behavioral Outcomes with Post-stroke Fluoxetine in Tanzania: The Phase II MAMBO Trial.,” *The American journal of tropical medicine and hygiene*, 106(3), pp. 970–978. Available at: https://doi.org/10.4269/ajtmh.21-0653.

Mathur, S. *et al.* (2019) “Improving Prehospital Stroke Services in Rural and Underserved Settings With Mobile Stroke Units,” 10(March), pp. 1–11. Available at: https://doi.org/10.3389/fneur.2019.00159.

Mcclelland, G. *et al.* (2015) “The challenges of conducting prehospital research : successes and lessons learnt from the Head Injury Transportation Straight to Neurosurgery,” pp. 663–664. Available at: https://doi.org/10.1136/emermed-2014-203870.

Meara, R.M.O., Ganas, U. and Hendrikse, C. (2022) “African Journal of Emergency Medicine Access to acute stroke care : A retrospective descriptive analysis of stroke patients ’ journey to a district hospital,” *African Journal of Emergency Medicine*, 12(4), pp. 366–372. Available at: https://doi.org/10.1016/j.afjem.2022.07.010.

Mellon, L. *et al.* (2013) “Can a media campaign change health service use in a population with stroke symptoms ? Examination of the fi rst Irish stroke awareness campaign,” pp. 1–5. Available at: https://doi.org/10.1136/emermed-2012-202280.

Mellon, L. *et al.* (2016) “Patient behaviour at the time of stroke onset : a cross-sectional survey of patient response to stroke symptoms,” pp. 396–402. Available at: https://doi.org/10.1136/emermed-2015-204806.

Minalloh, R.H. *et al.* (2022) “Pre-Hospital and In-Hospital Delay in Acute Ischemic Stroke Patients in Indonesia : A Multi-center Study,” 32(2), pp. 299–306.

Morris, D.L. *et al.* (2000) “Prehospital and Emergency Department Delays After Acute Stroke.” Available at: https://doi.org/10.1161/01.STR.31.11.2585.

Mould-millman, N., Sasser, S.M. and Wallis, L.A. (2013) “Prehospital Research in Sub-Saharan Africa: Establishing Research Tenets,” pp. 1304–1309. Available at: https://doi.org/10.1111/acem.12269.

Nagao, Y. *et al.* (2020) “Pre-Hospital Delay in Patients with Acute Ischemic Stroke in a Multicenter Stroke Registry : K-PLUS,” 29(00), pp. 1–7. Available at: https://doi.org/10.1016/j.jstrokecerebrovasdis.2020.105284.

Napon, C. *et al.* (2016) “Post-stroke epilepsy in Burkina Faso (West Africa).,” *Journal of the neurological sciences*, 368, pp. 47–48. Available at: https://doi.org/10.1016/j.jns.2016.06.038.

Nasreldein, A. *et al.* (2023) “Pre- and in-hospital delays in the use of thrombolytic therapy for patients with acute ischemic stroke in rural and urban Egypt.”

Nepal, G. *et al.* (2019) “Status of prehospital delay and intravenous thrombolysis in the management of acute ischemic stroke in Nepal,” *BMC Neurology*, 19(1), pp. 1–9. Available at: https://doi.org/10.1186/s12883-019-1378-3.

Nielsen, K. *et al.* (2012) “ASSESSMENT OF THE STATUS OF PREHOSPITAL CARE IN 13 LOW- AND MIDDLE-INCOME COUNTRIES,” pp. 381–389. Available at: https://doi.org/10.3109/10903127.2012.664245.

Niklasson, A., Herlitz, J. and Jood, K. (2019) “Socioeconomic disparities in prehospital stroke care,” pp. 1–9.

Nkoke, C. *et al.* (2015) “Stroke mortality and its determinants in a resource-limited setting: A prospective cohort study in Yaounde, Cameroon.,” *Journal of the neurological sciences*, 358(1–2), pp. 113–117. Available at: https://doi.org/10.1016/j.jns.2015.08.033.

Nkusi, A.E. *et al.* (2017) “Stroke Burden in Rwanda: A Multicenter Study of Stroke Management and Outcome.,” *World neurosurgery*, 106, pp. 462–469. Available at: https://doi.org/10.1016/j.wneu.2017.06.163.

Noubiap, J.J. and Nyaga, U.F. (2019) “A review of the epidemiology of atrial fibrillation in sub-Saharan Africa.,” *Journal of cardiovascular electrophysiology*, 30(12), pp. 3006–3016. Available at: https://doi.org/10.1111/jce.14222.

Ntep-Gweth, M. *et al.* (2010) “Atrial fibrillation in Africa: clinical characteristics, prognosis, and adherence to guidelines in Cameroon.,” *Europace : European pacing, arrhythmias, and cardiac electrophysiology : journal of the working groups on cardiac pacing, arrhythmias, and cardiac cellular electrophysiology of the European Society of Cardiology*, 12(4), pp. 482–487. Available at: https://doi.org/10.1093/europace/euq006.

Nutakki, A. *et al.* (2022) “Predictors of in-hospital and 90-day post-discharge stroke mortality in Lusaka, Zambia.,” *Journal of the neurological sciences*, 437, p. 120249. Available at: https://doi.org/10.1016/j.jns.2022.120249.

Ogbole, G.I. *et al.* (2015) “TIME OF PRESENTATION OF STROKE PATIENTS FOR CT IMAGING IN A NIGERIAN TERTIARY HOSPITAL,” 13(1), pp. 9–12.

Ojagbemi, A. *et al.* (2021) “Prevalence, predictors, and prognoses of prestroke neuropsychiatric symptoms at 3 months poststroke.,” *International psychogeriatrics*, 33(8), pp. 827–834. Available at: https://doi.org/10.1017/S1041610220003816.

Olum, S. *et al.* (2021) “Stroke Mortality Outcomes in Uganda.,” *Journal of stroke and cerebrovascular diseases : the official journal of National Stroke Association*, 30(5), p. 105661. Available at: https://doi.org/10.1016/j.jstrokecerebrovasdis.2021.105661.

Onwuegbuzie, G. A1*, Abdulai, F1, Obianozie, N. (2024) “Reasons for Delay in Presentation of Acute Stroke Patients in Teritary Health Centre in Abuja,” 10(July 2023), pp. 365–372. Available at: https://doi.org/10.36347/sasjm.2024.v10i05.015.

Ouali, S. *et al.* (2019) “Factors associated to adequate time in therapeutic range with oral vitamin K antagonists in Tunisia.,” *La Tunisie medicale*, 97(1), pp. 113–121.

Owolabi L.F1, N.M. (2012) “Stroke in Developing Countries: Experience at Kano, Northwestern Nigeria,” 7(1), pp. 9–14.

Owolabi, M. *et al.* (2017) “Stroke in Indigenous Africans, African Americans, and European Americans: Interplay of Racial and Geographic Factors.,” *Stroke*, 48(5), pp. 1169–1175. Available at: https://doi.org/10.1161/STROKEAHA.116.015937.

Owolabi, M.O. *et al.* (2019) “Randomized Trial of an Intervention to Improve Blood Pressure Control in Stroke Survivors.,” *Circulation. Cardiovascular quality and outcomes*, 12(12), p. e005904. Available at: https://doi.org/10.1161/CIRCOUTCOMES.119.005904.

Panagiotis Papapanagiotou a Nicoletta Iacovidou, Konstantinos Spengos, Theodoros Xanthos, Ioannis Zaganas c Afrodite Aggelina, Athanasios Alegakis, K.V. (2011) “Temporal Trends and Associated Factors for Pre- Hospital and In-Hospital Delays of Stroke Patients over a 16-Year Period : The Athens Study,” pp. 199–206. Available at: https://doi.org/10.1159/000321737.

Patel, M.D. *et al.* (2011) “Prehospital Notification by Emergency Medical Services Reduces Delays in Stroke Evaluation Findings From the North Carolina Stroke Care Collaborative,” pp. 2263–2268. Available at: https://doi.org/10.1161/STROKEAHA.110.605857.

Patel, V.B. *et al.* (2005) “Ischemic stroke in young HIV-positive patients in Kwazulu-Natal, South Africa.,” *Neurology*, 65(5), pp. 759–761. Available at: https://doi.org/10.1212/01.wnl.0000174434.00402.b5.

Peixoto, K.O., Eduardo, C. and Correia, R. (2017) “Factors associated with prehospital delay in acute stroke : systematic review Fatores associados ao atraso pré-hospitalar no acidente vascular encefálico agudo : revisão sistemática,” 8(1), pp. 14–25.

Per Wester, Johan Rådberg, Bo Lundgren, M.P. (1999) “Factors Associated With Delayed Admission to Hospital and In-Hospital Delays in Acute Stroke and TIA,” pp. 40–49.

Perkins, J.D. *et al.* (2020) “Prevalence, Characteristics and Risk Factors for Embolic Stroke of Undetermined Source in West and South Asia and North African Population Residing in Qatar.,” *Journal of stroke and cerebrovascular diseases : the official journal of National Stroke Association*, 29(5), p. 104666. Available at: https://doi.org/10.1016/j.jstrokecerebrovasdis.2020.104666.

Philip-Ephraim, E.E. *et al.* (2015) “Factors associated with prehospital delay among stroke patients in a developing African country,” *International Journal of Stroke*, 10(4), pp. E39–E39. Available at: https://doi.org/10.1111/ijs.12469.

Potisopha, W. *et al.* (2020) “Sex Differences in Prehospital Delay in Patients With Acute Stroke A Systematic Review,” *Journal of Cardiovascular Nursing*, 35(6), pp. E77–E88. Available at: https://doi.org/10.1097/JCN.0000000000000715.

Potisopha, W. *et al.* (2023) “Decision Delay Is a Significant Contributor to Prehospital Delay for Stroke Symptoms.” Available at: https://doi.org/10.1177/01939459221105827.

Pulvers, J.N. and Watson, J.D.G. (2017) “If Time Is Brain Where Is the Improvement in Prehospital Time after Stroke?,” 8(November). Available at: https://doi.org/10.3389/fneur.2017.00617.

Rajajee, V. and Saver, J. (2005) “Prehospital Care of the Acute Stroke Patient Acute Stroke : The Role of EMS,” pp. 74–80. Available at: https://doi.org/10.1053/j.tvir.2005.03.004.

Rakchue, P. and Poonphol, S. (2019) “Factor influencing Pre-hospital Delay among Acute Ischemic Stroke Patients in Rajavithi Hospital,” 18(November 2018), pp. 5–13.

Ramalan, M.A. *et al.* (2023) “Prevalence and trends of adult overweight and obesity in Nigeria - A systematic review and meta-analysis.,” *Nigerian journal of clinical practice*, 26(1), pp. 1–15. Available at: https://doi.org/10.4103/njcp.njcp_1903_21.

Regenhardt, R.W. *et al.* (2019) “Opportunities for intervention: stroke treatments, disability and mortality in urban Tanzania.,” *International journal for quality in health care : journal of the International Society for Quality in Health Care*, 31(5), pp. 385–392. Available at: https://doi.org/10.1093/intqhc/mzy188.

Reiff, T. and Michel, P. (2017) “Reasons and evolution of non-thrombolysis in acute ischaemic stroke,” pp. 219–226. Available at: https://doi.org/10.1136/emermed-2015-205140.

Revathi, S., Ms, K. and Shankar, V. (2023) “Factors Associated with Prehospital Delay in Patients with Acute Stroke in South India,” pp. 82–90. Available at: https://doi.org/10.4103/ijcm.ijcm.

Rice, D.R. *et al.* (2022) “Efficacy of Fluoxetine for Post-Ischemic Stroke Depression in Tanzania.,” *Journal of stroke and cerebrovascular diseases : the official journal of National Stroke Association*, 31(1), p. 106181. Available at: https://doi.org/10.1016/j.jstrokecerebrovasdis.2021.106181.

Roberts, M. and Hughes, G. (1998) “Recent advances in the acute management of ischaemic stroke,” (August).

Robinson, T.G. *et al.* (2013) “The face arm speech test : does it encourage rapid recognition of important stroke warning symptoms ?,” pp. 467–471. Available at: https://doi.org/10.1136/emermed-2012-201471.

Rosamond, W.D., Gorton, R.A. and Hinn, A.R. (no date) “Rapid Response to Stroke Symptoms : The Delay in Accessing Stroke Healthcare ( DASH ) Study,” pp. 45–51. Available at: https://doi.org/10.1111/j.1553-2712.1998.tb02574.x.

Rossnagel, K. (2004) “O ut-of-Hospital Delays in Patients With Acute Stroke,” (November). Available at: https://doi.org/10.1016/j.annemergmed.2004.06.019.

Rudd, M. *et al.* (2015) “A systematic review of stroke recognition instruments in hospital and prehospital settings,” pp. 1–5. Available at: https://doi.org/10.1136/emermed-2015-205197.

Ruiz, R.G. *et al.* (2017) “Response to Symptoms and Prehospital Delay in Stroke Patients . Is It Time to Reconsider Stroke Awareness Campaigns ?,” *Journal of Stroke and Cerebrovascular Diseases* [Preprint]. Available at: https://doi.org/10.1016/j.jstrokecerebrovasdis.2017.09.036.

Rukn, S. Al *et al.* (2019) “Stroke in the Middle-East and North Africa: A 2-year prospective observational study of stroke characteristics in the region-Results from the Safe Implementation of Treatments in Stroke (SITS)-Middle-East and North African (MENA).,” *International journal of stroke : official journal of the International Stroke Society*, 14(7), pp. 715–722. Available at: https://doi.org/10.1177/1747493019830331.

Sarfo, F. *et al.* (2018) “PINGS (Phone-Based Intervention Under Nurse Guidance After Stroke): Interim Results of a Pilot Randomized Controlled Trial.,” *Stroke*, 49(1), pp. 236–239. Available at: https://doi.org/10.1161/STROKEAHA.117.019591.

Sarfo, F.S. *et al.* (2015) “Trends in stroke admission and mortality rates from 1983 to 2013 in central Ghana.,” *Journal of the neurological sciences*, 357(1–2), pp. 240–245. Available at: https://doi.org/10.1016/j.jns.2015.07.043.

Sarfo, F.S., Akassi, J., Adamu, S., *et al.* (2020) “Frequency and factors linked to refractory hypertension among stroke survivors in Ghana.,” *Journal of the neurological sciences*, 415, p. 116976. Available at: https://doi.org/10.1016/j.jns.2020.116976.

Sarfo, F.S., Akassi, J., Agyei, M., *et al.* (2020) “Risk Factor Control in Stroke Survivors with Diagnosed and Undiagnosed Diabetes: A Ghanaian Registry Analysis.,” *Journal of stroke and cerebrovascular diseases : the official journal of National Stroke Association*, 29(12), p. 105304. Available at: https://doi.org/10.1016/j.jstrokecerebrovasdis.2020.105304.

Sarfo, F.S. *et al.* (2021) “Frequency and factors associated with post-stroke seizures in a large multicenter study in West Africa.,” *Journal of the neurological sciences*, 427, p. 117535. Available at: https://doi.org/10.1016/j.jns.2021.117535.

Sarfo, F.S., Ovbiagele, B., *et al.* (2022) “Differential associations between pre-diabetes, diabetes and stroke occurrence among West Africans.,” *Journal of stroke and cerebrovascular diseases : the official journal of National Stroke Association*, 31(11), p. 106805. Available at: https://doi.org/10.1016/j.jstrokecerebrovasdis.2022.106805.

Sarfo, F.S., Akassi, J., *et al.* (2022) “Long-term determinants of death after stroke in Ghana: Analysis by stroke types & subtypes.,” *Journal of stroke and cerebrovascular diseases : the official journal of National Stroke Association*, 31(9), p. 106639. Available at: https://doi.org/10.1016/j.jstrokecerebrovasdis.2022.106639.

Sarfo, F.S. and Ovbiagele, B. (2020) “Apparent Treatment Resistant Hypertension Among Stroke Survivors in Ghana.,” *Journal of stroke and cerebrovascular diseases : the official journal of National Stroke Association*, 29(12), p. 105401. Available at: https://doi.org/10.1016/j.jstrokecerebrovasdis.2020.105401.

Seremwe, F. *et al.* (2017) “Factors associated with hospital arrival time after the onset of stroke symptoms: A cross-sectional study at two teaching hospitals in Harare, Zimbabwe,” *Malawi Medical Journal*, 29(2), pp. 171–176. Available at: https://doi.org/10.4314/mmj.v29i2.18.

Shahbandi, A. *et al.* (2022) “Burden of stroke in North Africa and Middle East, 1990 to 2019: a systematic analysis for the global burden of disease study 2019.,” *BMC neurology*, 22(1), p. 279. Available at: https://doi.org/10.1186/s12883-022-02793-0.

Shaw, L. *et al.* (2014) “Paramedic Initiated Lisinopril For Acute Stroke Treatment ( PIL-FAST ): results from the pilot randomised controlled trial,” pp. 994–999. Available at: https://doi.org/10.1136/emermed-2013-202536.

Sheppard, J.P. *et al.* (2013) “The association between prehospital care and in-hospital treatment decisions in acute stroke : a cohort study,” pp. 1–7. Available at: https://doi.org/10.1136/emermed-2013-203026.

Sheppard, J.P. *et al.* (2016) “Prevalence and predictors of hospital prealerting in acute stroke : a mixed methods study,” pp. 482–488. Available at: https://doi.org/10.1136/emermed-2014-204392.

Shin, J. *et al.* (2023) “Trends in Prehospital Visits as a Cause of Delayed Admission in Korean Stroke Patients over a 10-Year Period : A National Health Insurance Claims Data Study,” 24(3).

Shpak, M. *et al.* (2019) “Driving stroke quality improvement at scale in EDs across a nationwide network of hospitals : strategies and interventions,” pp. 423–430. Available at: https://doi.org/10.1136/emermed-2018-208257.

Siddiqui, M. *et al.* (2008) “Factors delaying hospital arrival of patients with acute stroke,” pp. 178–182.

Smith, A.M. *et al.* (2011) “Paramedic decision making : prehospital thrombolysis and beyond,” pp. 2009–2012. Available at: https://doi.org/10.1136/emj.2009.083766.

Smyth, A. *et al.* (2021) “Renal Impairment and Risk of Acute Stroke: The INTERSTROKE Study.,” *Neuroepidemiology*, 55(3), pp. 206–215. Available at: https://doi.org/10.1159/000515239.

Sommer, P. *et al.* (2017) “Prehospital and intra-hospital time delays in posterior circulation stroke : results from the Austrian Stroke Unit Registry,” pp. 131–138. Available at: https://doi.org/10.1007/s00415-016-8330-x.

Soto-cámara, R. *et al.* (2021) “Factors related to prehospital time in caring for patients with stroke,” pp. 454–463.

Stead, L.G. *et al.* (2008) “Knowledge of signs , treatment and need for urgent management in patients presenting with an acute ischaemic stroke or transient ischaemic attack : a prospective study,” pp. 735–739. Available at: https://doi.org/10.1136/emj.2008.058206.

Sun, H. *et al.* (2024) “Global, Regional, and National Burdens of Stroke in Children and Adolescents From 1990 to 2019: A Population-Based Study.,” *Stroke*, 55(6), pp. 1543–1553. Available at: https://doi.org/10.1161/STROKEAHA.123.044827.

Talle, M.A. *et al.* (2023) “Clinical Profile of Patients with Hypertensive Emergency Referred to a Tertiary Hospital in the Western Cape Province of South Africa.,” *Current hypertension reviews*, 19(3), pp. 194–205. Available at: https://doi.org/10.2174/0115734021266958231101094556.

Tanne, D., Yaari, S. and Goldbourt, U. (1998) “Risk profile and prediction of long-term ischemic stroke mortality: a 21-year follow-up in the Israeli Ischemic Heart Disease (IIHD) Project.,” *Circulation*, 98(14), pp. 1365–1371. Available at: https://doi.org/10.1161/01.cir.98.14.1365.

Tataris, K.L., Mercer, M.P. and Govindarajan, P. (2015) “Prehospital aspirin administration for acute coronary syndrome ( ACS ) in the USA : an EMS quality assessment using the NEMSIS 2011 database,” pp. 1–6. Available at: https://doi.org/10.1136/emermed-2014-204299.

Teuschl, Y. and Ã, M.B. (2010) “Reviews Stroke education : discrepancies among factors influencing prehospital delay and stroke knowledge,” 5(June), pp. 187–208. Available at: https://doi.org/10.1111/j.1747-4949.2010.00428.x.

Tiu, C. (2022) “Pre-Hospital Delay in Acute Ischemic Stroke Care : Current Findings and Future Perspectives in a Tertiary Stroke Center.”

Togher, F.J., Davy, Z. and Siriwardena, A.N. (2012) “Patients ’ and ambulance service clinicians ’ experiences of prehospital care for acute myocardial infarction and stroke : a qualitative study,” pp. 942–949. Available at: https://doi.org/10.1136/emermed-2012-201507.

Tong, D. *et al.* (2012) “Times From Symptom Onset to Hospital Arrival in the Get With The Guidelines – Stroke Program 2002 to 2009 Temporal Trends and Implications,” pp. 1912–1917. Available at: https://doi.org/10.1161/STROKEAHA.111.644963.

Tran, J. and Mirzaei, M. (2011) “The population attributable fraction of stroke associated with high blood pressure in the Middle East and North Africa.,” *Journal of the neurological sciences*, 308(1–2), pp. 135–138. Available at: https://doi.org/10.1016/j.jns.2011.05.016.

Tshikwela, M.L., Londa, F.B. and Tongo, S.Y. (2015) “Stroke subtypes and factors associated with ischemic stroke in Kinshasa, Central Africa.,” *African health sciences*, 15(1), pp. 68–73. Available at: https://doi.org/10.4314/ahs.v15i1.9.

Ungerer, M.N. *et al.* (2020) “Factors affecting prehospital delay in rural and urban patients with stroke : a prospective survey-based study in Southwest Germany,” pp. 1–7.

Urimubenshi, G. *et al.* (2018) “Stroke care in Africa: A systematic review of the literature,” *International Journal of Stroke*, 13(8), pp. 797–805. Available at: https://doi.org/10.1177/1747493018772747.

V. V. Ashraf, M. Maneesh, R. Praveenkumar, K. Saifudheen, A.S.G. (2015) “Factors delaying hospital arrival of patients with acute stroke,” 18(2). Available at: https://doi.org/10.4103/0972-2327.150627.

Wannarong, T. and Nilanont, Y. (2019) “Factors Associated with Hospital Arrival Time in Acute Stroke,” pp. 1–7.

Williams, E.W. *et al.* (2009) “Delays in Presentations of Stroke Patients at the University Hospital of the Demoras en las Presentaciones de Pacientes con Accidente Cerebrovascular en el Hospital Universitario de West Indies,” 58(4).

Wubshet, A. *et al.* (2023) “Clinical characteristics and short-term outcomes of adult stroke patients admitted to Jimma Medical Center, Ethiopia: a prospective cohort study.,” *The Pan African medical journal*, 44, p. 49. Available at: https://doi.org/10.11604/pamj.2023.44.49.37588.

Xie, Z. *et al.* (2024) “Global impact of particulate matter on ischemic stroke.,” *Frontiers in public health*, 12, p. 1398303. Available at: https://doi.org/10.3389/fpubh.2024.1398303.

Yanagida, T. *et al.* (2014a) “Journal of Clinical Gerontology & Geriatrics Causes of prehospital delay in stroke patients in an urban aging society,” 5, pp. 7–11. Available at: https://doi.org/10.1016/j.jcgg.2014.02.001.

Yanagida, T. *et al.* (2014b) “Prehospital Delay and Stroke-related Symptoms.” Available at: https://doi.org/10.2169/internalmedicine.54.2684.

Yang, N. and Hartanto, Y.B. (2024) “Characteristics and reasons for delayed presentation in acute ischemic stroke : single ‑ centered study in Indonesia,” *The Egyptian Journal of Neurology, Psychiatry and Neurosurgery*, pp. 4–11. Available at: https://doi.org/10.1186/s41983-024-00838-4.

Yatsuya, H. *et al.* (2014) “Global trend in overweight and obesity and its association with cardiovascular disease incidence.,” *Circulation journal : official journal of the Japanese Circulation Society*, 78(12), pp. 2807–2818. Available at: https://doi.org/10.1253/circj.cj-14-0850.

YCASiu, TWWong, C.Cl. (1999) “Candidates for thrombolytic treatment in acute ischaemic stroke-where are our patients in Hong Kong ?,” (July), pp. 412–417.

Yeping Song, Fei Shen, Qing Dong, Liling Wang, J.M. (2023) “Prediction of Late Hospital Arrival in Patients with Mild and Rapidly Improving Acute Ischemic Stroke in a Rural Area of China,” (June), pp. 1119–1129.

Yoon, C.W. *et al.* (2022) “Comparisons of Prehospital Delay and Related Factors Between Acute Ischemic Stroke and Acute Myocardial Infarction,” *Journal of the American Heart Association*, 11(9). Available at: https://doi.org/10.1161/JAHA.121.023214.

Yuan, J. *et al.* (2023) “Articles Age and geographic disparities in acute ischaemic stroke prehospital delays in China : a cross-sectional study using national stroke registry data,” *The Lancet Regional Health - Western Pacific*, 33(118), p. 100693. Available at: https://doi.org/10.1016/j.lanwpc.2023.100693.

Yuko Tanaka, Makoto Nakajima, T.H. and M.U. (2009) “Factors Influencing Pre-Hospital Delay after Ischemic Stroke and Transient Ischemic Attack,” pp. 1739–1744. Available at: https://doi.org/10.2169/internalmedicine.48.2378.

Zewdie, A. *et al.* (2018) “Prospective assessment of patients with stroke in Tikur Anbessa Specialised Hospital, Addis Ababa, Ethiopia,” *African Journal of Emergency Medicine*, 8(1), pp. 21–24. Available at: https://doi.org/10.1016/j.afjem.2017.11.001.

Zhang, G. *et al.* (2018) “MMP Gene Polymorphisms, MMP-1 -1607 1G/2G, -519 A/G, and MMP-12 -82 A/G, and Ischemic Stroke: A Meta-Analysis.,” *Journal of stroke and cerebrovascular diseases : the official journal of National Stroke Association*, 27(1), pp. 140–152. Available at: https://doi.org/10.1016/j.jstrokecerebrovasdis.2017.08.021.

Zhang, R. *et al.* (2023) “Global Burden of Ischemic Stroke in Young Adults in 204 Countries and Territories.,” *Neurology*, 100(4), pp. e422–e434. Available at: https://doi.org/10.1212/WNL.0000000000201467.

Zhou, Y. *et al.* (2016) “Pre-hospital Delay after Acute Ischemic Stroke in Central Urban China : Prevalence and Risk Factors.” Available at: https://doi.org/10.1007/s12035-016-9750-4.
